# Spectral-Structural Decoupled Hypergraph Neural Network for *Fritillaria* Species Identification Using Hyperspectral Imaging

**DOI:** 10.3390/foods15152674

**Published:** 2026-07-29

**Authors:** Xincai Wang, Xiaoyu Fu, Kai Gao, Wenjie Liu, Wen Xiang, Yingjian Zhu, Yujie Lu, Chu Zhang

**Affiliations:** 1 Huzhou Institute for Food and Drug Control, Huzhou 313000, China; wangxc200307@163.com (X.W.); zxsz208wm@163.com (W.X.); 2 School of Information Engineering, Huzhou Normal University, Huzhou 313000, China; 2024388115@stu.zjhu.edu.cn (X.F.); 2024388116@stu.zjhu.edu.cn (K.G.); 2024388217@stu.zjhu.edu.cn (W.L.); 02896@zjhu.edu.cn (Y.L.); 3 Traditional Chinese Medical Hospital of Huzhou, Huzhou 313000, China; m13567239017@163.com

**Keywords:** hyperspectral imaging, *Fritillaria* species, Gramian Angular Difference Field, graph neural network, hypergraph neural network

## Abstract

*Fritillaria thunbergii* Miq. (ZBM) and *Fritillaria hupehensis* Hsiao et K.C. Hsia (HBBM) are two common medicinal and edible species of the genus *Fritillaria*, both possessing high economic and pharmacological value. Given their differences in pharmacological effects and economic value, rapid, nondestructive, and accurate identification of these two species is of practical significance. In this study, hyperspectral imaging (HSI) was used for the species identification of sliced *Fritillaria* samples. Based on the extracted one-dimensional spectra, conventional machine learning models, including Logistic Regression (LR), Support Vector Classification (SVC), and Extreme Gradient Boosting (XGBoost), as well as deep learning models, including one-dimensional Convolutional Neural Network (1D-CNN), one-dimensional Residual Network (1D-ResNet), Transformer, and CNN-Transformer, were first constructed. The one-dimensional spectra were further transformed into two-dimensional Gramian Angular Difference Field (GADF) images, followed by the construction of two-dimensional Convolutional Neural Network (2D-CNN) and two-dimensional Residual Network (2D-ResNet) models. To further explore structural relationships among spectral samples, graph-structured and hypergraph-structured data were constructed from both one-dimensional spectra and GADF images, and the corresponding Graph Convolutional Network (GCN) and Hypergraph Neural Network (HGNN) models were established. On this basis, a Spectral–Structural Decoupled Hypergraph Neural Network (SSD-HGNN) was proposed, in which deep spectral features extracted by 1D-ResNet were used as node attributes, while deep GADF image features extracted by 2D-ResNet were used to construct hyperedges. This design enabled decoupled fusion of one-dimensional spectral information and two-dimensional structural information within a hypergraph learning framework. The results showed that most models achieved satisfactory identification performance, demonstrating the feasibility of HSI for distinguishing ZBM and HBBM slices. SSD-HGNN achieved competitive overall performance, with accuracies of 0.9848, 0.9533, and 0.9441 on the training, validation, and test sets, respectively. Although SSD-HGNN did not achieve the highest test accuracy among all evaluated models, it achieved the highest validation accuracy and effectively integrated discriminative one-dimensional spectral features with two-dimensional GADF-based structural relationships. These results demonstrate that the proposed spectral–structural decoupled hypergraph learning strategy provides a competitive multimodal approach for rapid and nondestructive identification of *Fritillaria* species.

## 1. Introduction

*Fritillaria* refers to perennial herbaceous plants of the genus *Fritillaria* in the family Liliaceae and is widely cultivated in China [1,2]. Common species, such as *Fritillaria thunbergii* Miq. (ZBM) and *Fritillaria hupehensis* Hsiao et K.C. Hsia (HBBM), are medicinal and edible materials with considerable economic value [3]. Both ZBM and HBBM are mainly used in the form of dried bulbs, with HBBM primarily produced in Hubei Province, China and cultivated in different regions of the province, and ZBM mainly produced in Zhejiang Province, China and grown in different regions of the province. ZBM and HBBM exhibit both similarities and differences in morphological characteristics and chemical composition. Given their differences in pharmacological effects and economic value, accurate identification of ZBM and HBBM is important for medicinal-material quality control, market circulation management, and geographical-origin labeling. Fresh ZBM and HBBM may show more obvious differences in color, morphology, and surface appearance, and may therefore be easier to distinguish visually. However, in market circulation and practical quality-control applications, they are commonly processed into dried slices. During drying and slicing, some visible morphological and color differences may be weakened, making visual identification more difficult. Therefore, the present study focused on commercially prepared dried *Fritillaria* slices, for which rapid, nondestructive, and accurate instrumental identification is more practically needed. Although methods based on characteristic chemical constituents or specific markers can achieve accurate species authentication, they usually require complex sample preparation, high analytical cost, and relatively low detection efficiency, making them unsuitable for rapid screening of large-scale samples [4,5]. ZBM and HBBM have different main production areas, but potential adulteration or substitution may still occur during downstream processing and commercial circulation rather than at the cultivation site. After harvesting, *Fritillaria* bulbs are commonly processed into dried slices, packaged, transported, and traded through wholesale and retail channels. During these processes, the original geographical information may become less obvious, and the visual differences between dried slices from different sources may be weakened. Because the market price of ZBM is generally higher than that of HBBM, lower-value HBBM slices may be intentionally mixed with or substituted for ZBM in commercial circulation. Therefore, developing a rapid, nondestructive, and accurate method for distinguishing ZBM from HBBM is of practical significance for adulteration detection, quality control, and market regulation.

For the identification of species and geographical origin of ZBM and HBBM slices, developing a rapid and nondestructive detection technique capable of high-throughput analysis of individual slices is of practical significance. Hyperspectral imaging (HSI) is a widely studied rapid and nondestructive detection technique that can simultaneously capture both spatial and spectral information from tested samples, and it has been extensively applied to quality evaluation and identification of foods, traditional Chinese medicinal materials, and medicinal and edible materials [6,7,8]. The spatial-spectral acquisition capability of HSI enables simultaneous detection and accurate sorting of large-scale ZBM and HBBM slice samples. This approach can reduce errors and uncertainties associated with sampling-based measurements and avoid several limitations of wet-chemical methods, such as complex sample preparation, destructive procedures, and reliance on chemical reagents. Although HSI requires relatively high initial equipment investment, it can acquire spectral-spatial information from multiple samples in a single imaging process and is therefore advantageous for high-throughput screening. In large-scale quality-control scenarios, this batch detection capability may reduce labor demand and per-sample analytical cost compared with repeated wet-chemical analyses.

A common strategy for hyperspectral data analysis is to use the mean spectrum of all pixels within the region of interest (ROI) of each sample, which can substantially reduce data dimensionality. However, the high dimensionality of hyperspectral data remains a major challenge in data analysis and modeling. Machine learning methods have been widely used for hyperspectral data analysis. Common algorithms, such as partial least squares (PLS), support vector machine (SVM), and random forest (RF), have been extensively applied in various studies and are regarded as representative conventional modeling methods [9,10,11]. In recent years, deep learning has emerged as a powerful approach and has gradually been widely adopted in spectral data analysis [12,13,14]. Owing to its strong flexibility, deep learning can be applied to multiple stages of spectral analysis, including preprocessing, regression and classification modeling, feature extraction and feature selection, data generation, and transfer learning, and has therefore become one of the mainstream approaches in spectral data analysis [13,15]. Among these applications, deep learning-based regression and classification modeling are the most widely used directions in spectral analysis. In addition, Transformer-based architectures have recently been introduced into spectral and hyperspectral data analysis because of their ability to model long-range dependencies. Therefore, Transformer and CNN-Transformer models were also included in this study as additional deep learning baselines to provide a more comprehensive comparison with convolutional, residual, graph-based, and hypergraph-based models.

Owing to the flexibility of deep learning models, increasing efforts have been devoted to modifying basic deep learning architectures or exploring new deep learning methods to more effectively extract latent features from spectral data. Graph Neural Networks (GNNs) are a class of deep learning frameworks designed for graph-structured data, enabling representation learning in non-Euclidean domains. Their effectiveness largely depends on appropriate graph construction and efficient node feature propagation. Graph Convolutional Networks (GCNs), as a representative form of GNNs, aggregate information from neighboring nodes through graph convolution operations, thereby enabling local structural perception and parameter sharing. In recent years, GCNs have been widely applied to hyperspectral image analysis [16] and have also been gradually extended to standalone one-dimensional (1D) spectral data analysis [17,18,19,20]. However, conventional GNNs usually connect two nodes with an edge and mainly characterize pairwise relationships among samples, making it difficult to fully represent latent high-order associations among multiple samples. In contrast, Hypergraph Neural Networks (HGNNs) connect multiple nodes through hyperedges, allowing more complex multi-node relationships and high-order structural dependencies to be modeled [21,22,23]. HGNNs have recently attracted increasing attention in hyperspectral image analysis [24,25]. However, to the best of our knowledge, studies applying HGNNs to standalone 1D spectral data analysis remain limited.

One-dimensional deep learning models are typically applied directly to 1D spectral data, with the advantage that each wavelength corresponds to specific chemical bond information, allowing the model to fully retain the features of the original spectral sequence. However, the high dimensionality and complex nonlinear relationships in 1D spectra may limit the model’s ability to capture deep spectral features. To further enhance feature representation and capture inter-wavelength relationships, transforming 1D spectra into two-dimensional (2D) images for input to established 2D deep learning models has been proposed as a promising strategy [26,27,28,29]. The Gramian Angular Difference Field (GADF) is a widely used method for mapping one-dimensional signals into two-dimensional images, which has shown effectiveness in spectral data analysis [26,28,30]. By converting 1D spectra into 2D GADF images, the absolute positions of wavelengths are preserved, and spectral variations are represented as corresponding local pixel differences in the image, thereby enhancing both local and global feature representation of the spectra.

Based on the above considerations, this study employed hyperspectral imaging for the rapid and nondestructive identification of ZBM and HBBM slices and further proposed a Spectral-Structural Decoupled Hypergraph Neural Network (SSD-HGNN). Instead of simply concatenating 1D spectral features and 2D GADF image features, SSD-HGNN assigns different functional roles to these two modalities within a hypergraph learning framework. Specifically, a one-dimensional Residual Network (1D-ResNet) is first used to extract deep spectral features from the original spectra, which are then used as node attributes to preserve the direct discriminative information embedded in the spectral sequence. Meanwhile, the 1D spectra are transformed into 2D GADF images, and a 2D-ResNet is used to extract deep structural features from the GADF images for hyperedge construction. In this way, the hyperedges represent high-order similarities among samples in the two-dimensional inter-wavelength structural feature space. Through this decoupled fusion strategy between node attributes and hyperedge topology, SSD-HGNN can jointly exploit the sequential discriminative information of 1D spectra and the structural relationship information of 2D GADF images, thereby enabling collaborative modeling of multimodal spectral features and high-order inter-sample relationships.

To systematically validate the effectiveness of the proposed method, the main objectives of this study were as follows:

(1) to construct conventional machine learning models, including Logistic Regression (LR), Extreme Gradient Boosting (XGBoost), and Support Vector Classification (SVC), as well as deep learning models, including one-dimensional Convolutional Neural Network (1D-CNN), 1D-ResNet18, and 1D-ResNet34, Transformer, and CNN-Transformer, based on 1D spectra for the identification of ZBM and HBBM;

(2) to transform 1D spectra into 2D GADF images and establish 2D-CNN, 2D-ResNet18, and 2D-ResNet34 models to evaluate the effectiveness of image-based spectral representation;

(3) to construct GCN and HGNN models based on 1D spectra and 2D GADF images, respectively, to investigate the roles of graph and hypergraph structures in modeling spectral sample relationships;

(4) to develop the proposed SSD-HGNN by integrating 1D spectral features and 2D GADF structural features, and to compare it systematically with the above models to evaluate the effectiveness of spectral-structural decoupled hypergraph fusion for ZBM and HBBM species identification.

## 2. Materials and Methods

### 2.1. Sample Preparation

The ZBM and HBBM samples used in this study were collected from Jinhua, Zhejiang Province, and Enshi Tujia and Miao Autonomous Prefecture, Hubei Province, China, respectively. All samples were commercially prepared dried *Fritillaria* slices. For each species, the samples were obtained from multiple batches rather than from a single bulb or a single batch. This multi-batch sample composition introduced within-class variability caused by differences in raw materials, processing, and batch-level variation, making the dataset closer to practical commercial circulation and quality-control scenarios. Because the samples were obtained as commercially circulated dried slices rather than collected from individually tracked plants, the exact numbers of original bulbs and independent plants were unavailable.

**Figure 1:**
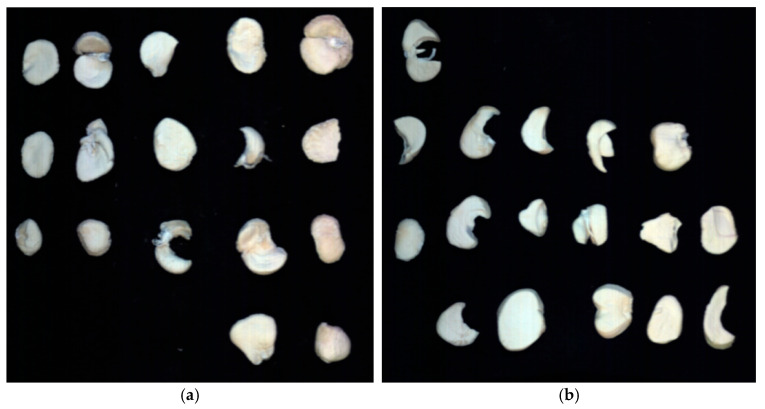
Pseudocolor images of ZBM (**a**) and HBBM (**b**).

The samples were imaged in batches, yielding 201 hyperspectral images for ZBM and 187 hyperspectral images for HBBM. Each complete and valid dried slice segmented from these hyperspectral images was treated as an individual sample for spectral extraction, dataset partitioning, and species identification. Each slice was assigned to only one of the training, validation, or test sets, and no complete slice or extracted spectrum was shared across different subsets. Figure 1 shows representative pseudocolor images of ZBM and HBBM slices.

During sample selection, slices with incomplete morphology or severe overlap were excluded. Because ZBM and HBBM differed in bulb size, slicing condition, slice integrity, and the number of valid regions of interest obtained after image segmentation, the final numbers of qualified slices were not exactly the same between the two species. Nevertheless, both classes contained a large number of valid samples, and the overall class distribution was relatively balanced. The training, validation, and test sets were divided at the slice level using a stratified strategy to maintain similar class distributions across different subsets. Table 1 summarizes the sample distribution of ZBM and HBBM.

### 2.2. Hyperspectral Image Acquisition and Spectral Data Extraction

#### 2.2.1. Hyperspectral Image Acquisition

**Figure 2:**
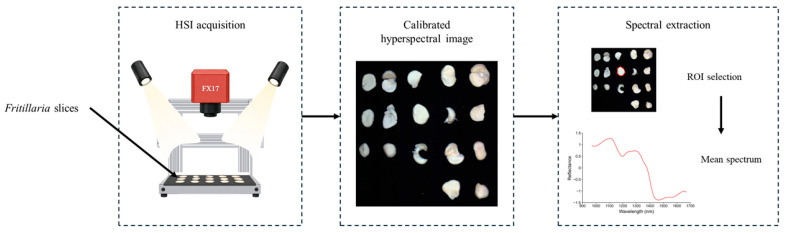
Workflow of hyperspectral imaging acquisition and spectral extraction of *Fritillaria* slices. The red dashed line represents the selected ROI area.

In this study, hyperspectral images of *Fritillaria* slices were acquired using a laboratory-based near-infrared hyperspectral imaging system. The system was equipped with an FX17 hyperspectral camera (Spectral Imaging Ltd., Oulu, Finland), which covered a spectral range of 900–1700 nm with 224 spectral bands. Illumination was provided by two sets of halogen lamps, each consisting of three 35 W bulbs. The lamps were symmetrically positioned on both sides of the camera, and their angles were adjusted to ensure uniform illumination of the sample area beneath the camera lens. The hyperspectral camera and halogen lamps were mounted on a LabScanner platform equipped with a motorized translation stage. Image acquisition was controlled using Lumo-Scanner 2020 software (Spectral Imaging Ltd., Oulu, Finland). During image acquisition, the *Fritillaria* slices were placed separately on the moving platform to avoid contact or overlap between samples. A white reference image obtained from a Teflon board and a dark reference image were used for image calibration. After raw hyperspectral image acquisition, reflectance calibration was performed according to Equation (1):

(1) $$R_{img}=\frac{I_{raw}-I_{dark}}{I_{white}-I_{dark}}\;,$$

where $R_{img}$ denotes the calibrated hyperspectral image, $I_{raw}$ denotes the raw hyperspectral image, $I_{white}$ denotes the white reference image, and $I_{dark}$ denotes the dark reference image. The workflow of hyperspectral image acquisition and spectral extraction is shown in Figure 2.

#### 2.2.2. Spectral Data Extraction

As shown in Figure 1, there was a clear contrast between the *Fritillaria* slices and the background. In this study, each complete Fritillaria slice was treated as a ROI, and the mean spectrum of each slice was extracted for subsequent modeling. The spectral extraction procedure was performed as follows.

(1) Because the spectral responses of the background and the samples differed substantially, the grayscale image at the 100th spectral band was selected to generate a mask. Firstly, threshold-based binarization was applied to the grayscale image, assigning pixels below the threshold a value of 0 and pixels above the threshold a value of 1, thereby producing a binary image. Morphological erosion and dilation operations were then performed to remove small noisy pixels. The final binary image contained background regions with a value of 0 and sample regions with a value of 1.

(2) The binary mask was multiplied by the original hyperspectral data band by band at the corresponding pixel positions, setting the background reflectance to 0 and retaining only the spectral information of the *Fritillaria* slices. Based on the background-removed hyperspectral images, connected-component analysis was performed on the binary image to label each valid slice region. For each valid *Fritillaria* slice region, a rectangular region containing the complete slice was cropped using the centroid of the connected component as the center.

(3) During spectral extraction, the reflectance values of all pixels within each slice region were averaged at each spectral band to obtain the mean reflectance of the sample at the corresponding band. The mean reflectance values from all bands were then combined to generate the mean spectrum of each sample across the spectral range of the imaging system.

Due to the response characteristics of the hyperspectral imaging system, the spectral bands at both ends of the acquired spectra contained relatively high noise. Therefore, the first 13 bands and the last 11 bands were removed, and the remaining 200 bands, covering the spectral range of 980–1680 nm, were retained for subsequent analysis. The extracted spectra were further preprocessed using moving average smoothing with a five-point window combined with standard normal variate (SNV) transformation. Moving average smoothing was mainly used to reduce spectral noise, whereas SNV was applied to reduce non-chemical interference caused by sample scattering effects and baseline drift. The preprocessed spectra were used for subsequent modeling analysis.

### 2.3. GADF Image Generation

To fully exploit the intrinsic relationships between different spectral bands in the 1D spectral sequences, the GADF method was employed to convert the spectral data into two-dimensional image representations in this study [31]. This method maps the 1D spectral responses into polar coordinates using angular encoding and subsequently constructs a matrix of inter-band angular differences, thereby transforming the original sequential spectral information into 2D image features with spatial texture structure [26,28,30].

To adapt the GADF matrices for subsequent two-dimensional deep learning models, the matrices were further mapped into RGB pseudocolor images. Specifically, the values of each GADF matrix were first linearly scaled to the [0, 1] interval, and a rainbow colormap was applied to generate three-channel images. All GADF images were resized to 200 × 200 pixels to ensure consistent input dimensions. Consequently, each spectral sample in the training, validation, and test sets was converted into a corresponding GADF image, maintaining a one-to-one correspondence between the image samples, class labels, and the original spectral samples.

**Figure 3:**
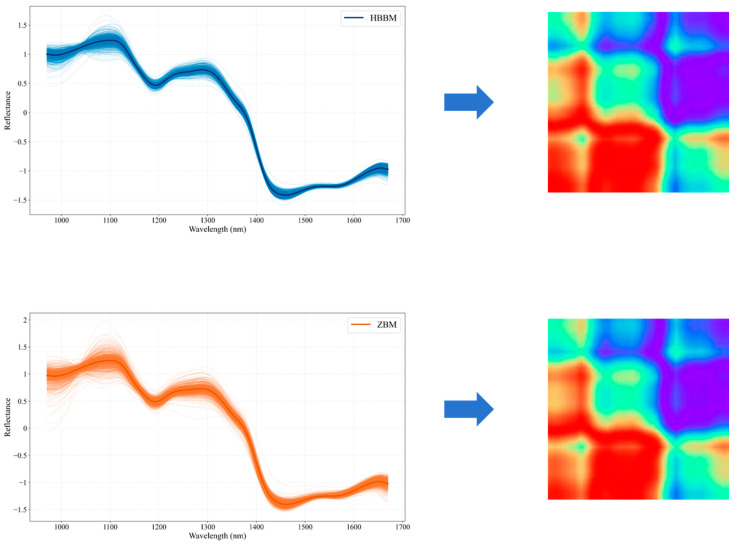
Preprocessed 1D spectral curves of two *Fritillaria* varieties and the GADF image corresponding to one of the spectra.

Figure 3 illustrates the 1D spectral curves of two *Fritillaria* varieties and the GADF image corresponding to one of the spectra after preprocessing. The differences between the varieties in the 1D spectral curves primarily manifest as variations in local band response intensity, curve trends, and adjacent band fluctuation patterns. After GADF encoding, these one-dimensional sequence differences are further transformed into color distributions, texture orientations, and local structural variations in the two-dimensional images. Compared with using raw 1D spectral sequences directly, GADF images can more intuitively depict inter-band relationships in a spatial format, enabling 2D convolutional networks to simultaneously capture local texture features and global inter-spectral associations. This enhanced feature representation provides richer inputs for subsequent 2D-CNN, 2D-ResNet18, and 2D-ResNet34 models for classification and identification.

### 2.4. Data Analysis Methods

#### 2.4.1. Conventional Machine Learning Methods

Conventional machine learning methods have been widely applied to spectral data analysis, and their feasibility and effectiveness have been demonstrated in numerous studies. In this study, LR [32], SVC [33], and XGBoost [34] were used as conventional machine learning baseline models to construct classification models for ZBM and HBBM. Their performance was compared with that of the proposed model. The main hyperparameters of these conventional machine learning models were determined based on commonly used settings in previous spectral-analysis studies, preliminary experiments, model convergence behavior, and validation-set performance. The validation set was used for model selection and hyperparameter adjustment, whereas the test set was used only for final performance evaluation.

LR is a commonly used linear classification model that maps the output of a linear model to class probabilities through the Sigmoid function. It is frequently used as a baseline classifier for high-dimensional feature data. In this study, to reduce the influence of differences in the numerical ranges of spectral variables on model convergence, the input spectral features were standardized using StandardScaler before model training. To avoid information leakage, StandardScaler was fitted only on the training set, and the learned scaling parameters were then applied to the validation and test sets. L2 regularization was adopted in the LR classifier to reduce the risk of overfitting in high-dimensional spectral data. The regularization strength was set to 1.0, and the maximum number of iterations was set to 5000 to ensure sufficient model convergence.

SVC is the classification form of SVM. It constructs a discriminative hyperplane by maximizing the classification margin and is well suited to high-dimensional spectral classification tasks. Considering that the spectral differences among sample classes may exhibit nonlinear distributions, a radial basis function (RBF) kernel was used in this study to achieve nonlinear feature mapping. The penalty coefficient C was set to 100,000, and the kernel coefficient gamma was set to 0.1. Here, C controls the trade-off between the classification margin and misclassified samples, whereas gamma determines the local response range of the RBF kernel.

XGBoost is an ensemble learning method based on gradient-boosted decision trees. It improves classification performance by iteratively fitting residuals and is suitable for modeling spectral features with nonlinear relationships. In this study, binary logistic regression was used as the objective function, and logloss was used as the training evaluation metric. The number of decision trees was set to 300, the learning rate was set to 0.05, and the maximum tree depth was set to 3. To improve model generalization, the sample subsampling ratio was set to 0.8, and the feature subsampling ratio was set to 0.8. In addition, the L2 regularization coefficient was set to 1.0, the tree construction method was set to hist, and the model was trained using parallel processing across all available CPU cores to accelerate computation.

#### 2.4.2. Deep Learning Methods

Deep learning methods have been widely applied to spectral data analysis. In this study, commonly used deep learning models, including CNN, Transformer, CNN-Transformer, ResNet, and GCN, were constructed based on the spectral data of ZBM and HBBM as baseline models for species identification. The main hyperparameters of the deep learning models were determined based on commonly used settings in previous spectral-analysis studies, preliminary experiments, model convergence behavior, and validation-set performance. The training set was used for model parameter optimization, the validation set was used for model selection and hyperparameter adjustment, and the test set was used only for final performance evaluation.

CNNs are among the most commonly used and fundamental deep learning models in spectral data analysis [35]. A CNN generally consists of convolutional layers, pooling layers, activation functions, and fully connected layers. Through feature learning and feature extraction, CNNs can perform classification or regression tasks using input data. In this study, two customized CNN models were designed for the extracted 1D spectral data and the corresponding 2D GADF images of ZBM and HBBM, namely 1D-CNN and 2D-CNN.

**Figure 4:**
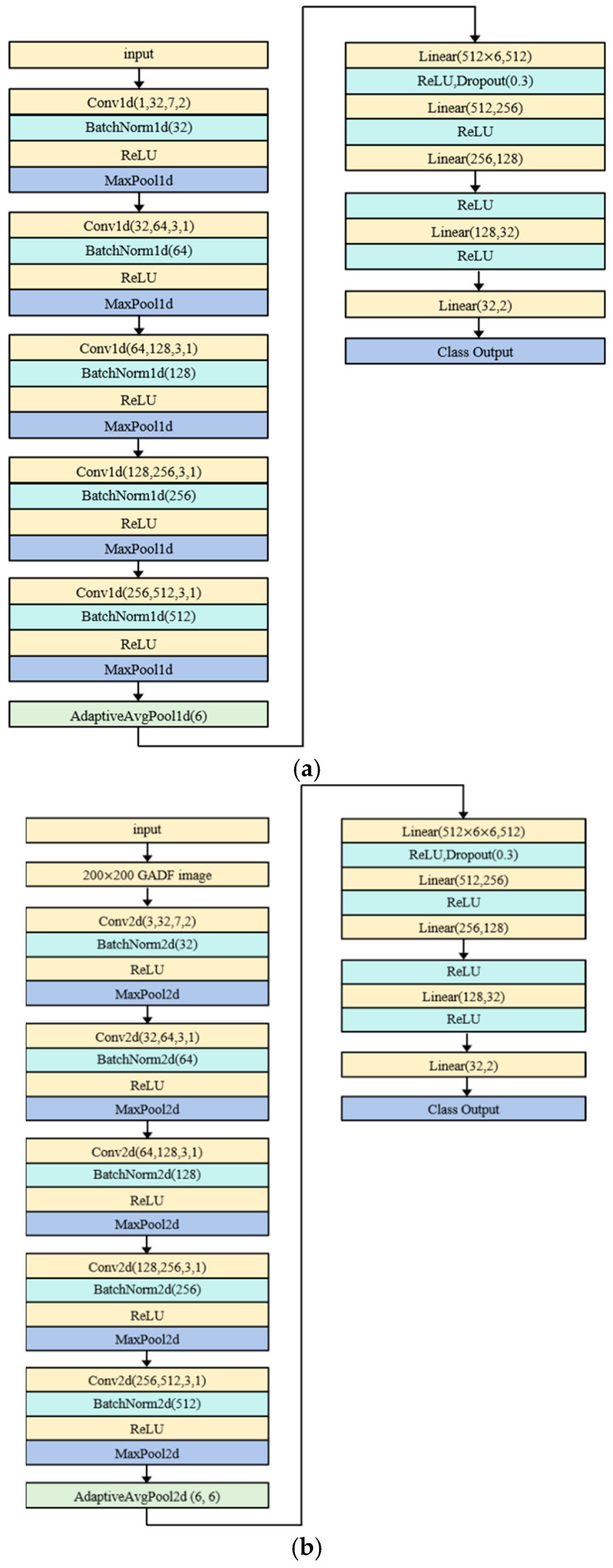
CNN model architectures for classification of ZBM and HBBM. (**a**) Framework of 1D-CNN based on 1D spectral sequences; (**b**) Framework of 2D-CNN based on GADF images.

The 1D-CNN was designed to directly perform convolutional modeling on 1D spectral sequences, enabling the model to extract local response features between adjacent bands and multilevel spectral patterns. The input data were represented as single-channel 1D spectral sequences. The feature extraction module consisted of five 1D convolutional blocks with channel numbers of 32, 64, 128, 256, and 512, respectively. The first layer used a 1D convolution with a kernel size of 7 for initial feature extraction, whereas the subsequent convolutional layers used 1D convolutions with a kernel size of 3 to further extract local spectral features. Each convolutional block included Conv1d, BatchNorm1d, ReLU, and MaxPool1d operations. The convolutional features were mapped to a fixed length using AdaptiveAvgPool1d (6) and then fed into a fully connected classifier. The classifier contained multiple fully connected layers with dimensions of 512, 256, 128, and 32, and finally output the two-class classification results. During training, the Adam optimizer was used, Cross-Entropy Loss was adopted as the loss function, the number of training epochs was set to 300, the batch size was set to 32, the learning rate was set to 0.001, and the weight decay coefficient was set to 1 × 10^−4^. The architecture of 1D-CNN is shown in Figure 4a.

The 2D-CNN was constructed using GADF images converted from the original 1D spectra as input, allowing the model to extract inter-spectral relationship features and image texture features through 2D convolutional structures. The input image size was uniformly set to 200 × 200 × 3. The feature extraction module consisted of five 2D convolutional blocks with channel numbers of 32, 64, 128, 256, and 512, respectively. The first layer used a 7 × 7 convolutional kernel for initial feature extraction, whereas the subsequent convolutional layers used 3 × 3 convolutional kernels to further extract local texture information. Each convolutional block included Conv2d, BatchNorm2d, ReLU, and MaxPool2d operations. The convolutional features were mapped to a fixed size using AdaptiveAvgPool2d ((6, 6)) and then fed into a fully connected classifier. The classifier contained multiple fully connected layers with dimensions of 512, 256, 128, and 32, and finally output the two-class classification results. During training, the Adam optimizer was used, Cross-Entropy Loss was adopted as the loss function, the number of training epochs was set to 300, the batch size was set to 16, the learning rate was set to 0.001, and the weight decay coefficient was set to 1 × 10^−4^. The architecture of 2D-CNN is shown in Figure 4b.

Transformer [36] was adopted as an attention-based deep learning baseline model for global spectral feature modeling. Unlike CNNs, which mainly extract local spectral patterns through convolutional kernels, Transformer relies on the self-attention mechanism to directly model interactions among different spectral positions. This enables the model to capture long-range dependencies and global contextual information within the spectral sequence. In this study, the input 1D spectral sequence was first projected into an embedding space and then processed by Transformer encoder layers for feature extraction. The learned global spectral representation was subsequently fed into fully connected layers to output the final classification result. The model was optimized using the Adam optimizer with Cross-Entropy Loss as the objective function. The number of attention heads was set to 4, the number of encoder layers was set to 2, the batch size was set to 32, the learning rate was set to 0.0001, and the weight decay coefficient was set to 1 × 10^−4^.

CNN-Transformer [37] was used as another hybrid deep learning baseline model to combine local spectral representation learning with global attention-based modeling. Specifically, CNN layers were first applied to extract local spectral features and reduce local redundancy in the spectral sequence. The extracted feature representations were then processed by Transformer encoder layers to capture long-range dependencies and global contextual interactions among spectral features. Finally, the output features were fed into fully connected layers for binary classification. This model was included to evaluate whether combining local convolutional modeling and global self-attention modeling could further improve ZBM and HBBM species identification. The model was optimized using the Adam optimizer with Cross-Entropy Loss as the objective function. The number of attention heads was set to 4, the number of encoder layers was set to 2, the batch size was set to 32, the learning rate was set to 0.0001, and the weight decay coefficient was set to 1 × 10^−4^.

ResNets introduce residual connections into conventional CNN architectures to alleviate gradient vanishing and network degradation during deep network training. ResNet-based models have been widely used in spectral data analysis because they can learn deeper and more discriminative features from spectral data [38]. In this study, ResNet18 and ResNet34 were adapted to both 1D spectral sequences and 2D GADF images, resulting in four models: 1D-ResNet18, 1D-ResNet34, 2D-ResNet18, and 2D-ResNet34. All ResNet-based feature extractors were trained using only the training samples and their labels. The validation set was used only for model selection, and the test set was used only for final performance evaluation. Validation and test labels were not used during feature extractor training or parameter optimization.

The 1D-ResNet18 model applied residual learning to one-dimensional spectral sequence modeling, enabling the network to extract local spectral patterns and cross-band features while mitigating gradient degradation in deeper layers. The model accepted single-channel 1D spectral sequences as input and employed a hierarchical residual architecture with four stages. The residual block configuration was [2, 2, 2, 2], corresponding to the number of residual blocks in the four successive stages. Each residual block consisted of two one-dimensional convolutional layers with identity connections to enhance training stability. Global spectral features were aggregated using adaptive average pooling, and the final binary classification was produced by fully connected layers. During training, the model was optimized using the Adam optimizer with Cross-Entropy Loss as the objective function. The training was conducted for 300 epochs with a batch size of 32, a learning rate of 0.0001, and a weight decay coefficient of 1 × 10^−4^.

The 1D-ResNet34 model was constructed as a deeper residual network than 1D-ResNet18 to enhance deep spectral feature extraction. It used the same single-channel 1D spectral input form, but the residual stages followed a block configuration of [3, 4, 6, 3]. The larger number of residual blocks increased the network depth and enabled 1D-ResNet34 to learn more complex local spectral structures and cross-band association features. Global spectral features were aggregated using adaptive average pooling, and the final binary classification was performed using fully connected layers. During training, the model was optimized using the Adam optimizer with Cross-Entropy Loss as the objective function. The training was conducted for 300 epochs with a batch size of 32, a learning rate of 0.0001, and a weight decay coefficient of 1 × 10^−4^.

The 2D-ResNet18 model applied residual learning to the classification of two-dimensional GADF images, enabling the network to extract image texture features and inter-spectral relationship features derived from the original spectral sequences. The model accepted RGB GADF images with a size of 200 × 200 × 3 as input and employed a four-stage residual architecture for hierarchical feature extraction. The residual block configuration was [2, 2, 2, 2], corresponding to the number of residual blocks in the four successive stages. Each residual block consisted of two two-dimensional convolutional layers with identity connections to improve training stability and alleviate network degradation. Global image features were aggregated using adaptive average pooling, and the final binary classification was produced by fully connected layers. During training, the model was optimized using the Adam optimizer with Cross-Entropy Loss as the objective function. The training was conducted for 300 epochs with a batch size of 32, a learning rate of 0.0001, and a weight decay coefficient of 1 × 10^−4^.

The 2D-ResNet34 model was constructed as a deeper residual network than 2D-ResNet18 to enhance deep feature extraction from GADF images. It used the same RGB GADF image input form with a size of 200 × 200 × 3, but adopted a residual block configuration of [3, 4, 6, 3] across the four successive stages, compared with 2D-ResNet18. The increased number of residual blocks resulted in a deeper network architecture, allowing 2D-ResNet34 to learn more complex texture patterns and inter-spectral association features from the GADF images. Global image features were aggregated using adaptive average pooling, and the final binary classification was performed using fully connected layers. During training, the model was optimized using the Adam optimizer with Cross-Entropy Loss as the objective function. The training was conducted for 300 epochs with a batch size of 32, a learning rate of 0.0001, and a weight decay coefficient of 1 × 10^−4^.

GCNs are a type of GNN that extend convolutional operations to graph-structured data, enabling the model to jointly learn node attributes and graph topological information [16,17]. In this study, GCNs were used as graph-based deep learning baseline models to evaluate the effectiveness of modeling pairwise similarity relationships among *Fritillaria* samples.

In the GCN modeling process, each *Fritillaria* slice sample was defined as a node in the graph. Let the node feature matrix of all samples be:

(2) $$X\in R^{N\times d}\;,$$

where N denotes the number of samples and d denotes the dimension of the node features. The graph structure was constructed using a K-nearest neighbor (KNN) strategy based on cosine similarity. For each node, the K most similar nodes were selected as its neighbors. After the adjacency matrix was obtained, it was symmetrized to construct an undirected graph, and self-loops were added to preserve the information of each node itself. The adjacency matrix was then normalized as follows:

(3) $$\hat{A}=D^{-\frac{1}{2}}AD^{-\frac{1}{2}}\;,$$

where A denotes the symmetrized adjacency matrix with self-loops, and D denotes the corresponding degree matrix. The normalized adjacency matrix $\hat{A}$ was used for graph feature propagation.

In this study, a two-layer GCN was adopted for classification. The first graph convolutional layer mapped the input node features into a hidden feature space, and the second graph convolutional layer mapped the hidden features into the class space to generate the final classification output. The feature propagation process of the GCN can be expressed as:

(4) $$H^{\left(l+1\right)}=\sigma (\hat{A}H^{\left(l\right)}W^{\left(l\right)})\;,$$

where $H^{\left(l\right)}$ denotes the node representation at the l-th layer, $W^{\left(l\right)}$ denotes the trainable weight matrix, and σ(·) denotes the nonlinear activation function. In the implemented model, the hidden dimension was set to 256, and ReLU was used as the activation function. The model was optimized using the Adam optimizer with Cross-Entropy Loss as the objective function. The learning rate was set to 0.0003 the weight decay coefficient was set to 0.0001.

The 1D-GCN model was constructed based on the preprocessed 1D spectral features of ZBM and HBBM. In this model, each *Fritillaria* slice sample was treated as a graph node, and its preprocessed 1D spectrum was used as the initial node attribute. Before graph construction and model training, the spectral features were standardized using StandardScaler to reduce the influence of differences in variable scales. The StandardScaler was fitted only on the training samples, and the same scaling parameters were applied to validation or test samples before graph construction and inference. Cosine similarity was then calculated between spectral feature vectors, and a KNN graph was constructed by selecting the most similar neighboring samples for each node. In this study, K was set to 5 for KNN-based graph construction. This value was selected according to preliminary validation experiments to balance local similarity preservation and graph connectivity. For validation and test evaluation, a reference-set-based graph inference strategy was adopted. Specifically, the validation or test samples were inserted into the graph as unlabeled nodes together with the training samples, and predictions were evaluated only on the validation or test nodes. During this process, the labels of validation and test samples were strictly masked and were not used for graph construction, loss calculation, parameter optimization, or model training. The graph structure was constructed only according to feature similarity among samples. Therefore, this strategy did not introduce label leakage. This design also reflects a practical deployment scenario, in which a newly collected *Fritillaria* slice is not classified by constructing an isolated graph by itself, but is inserted into an existing reference graph and connected to labeled reference samples according to feature similarity before inference. Through graph convolutional propagation, the 1D-GCN model aggregated information from spectrally similar samples and produced the final binary classification results for ZBM and HBBM.

The 2D-GCN model was constructed based on the GADF image representation converted from the original 1D spectra. In this model, each *Fritillaria* slice sample was also treated as a graph node. The corresponding GADF image of each sample was flattened into a one-dimensional feature vector and used as the initial node attribute. This conversion allowed the GADF image representation to be incorporated into the node feature matrix required by the GCN model. Similar to 1D-GCN, cosine similarity was calculated between the flattened GADF feature vectors, and a KNN graph was constructed to describe pairwise relationships among samples in the GADF feature space. The adjacency matrix was symmetrized, self-loops were added, and the normalized adjacency matrix was used for graph convolutional propagation. Compared with 1D-GCN, which models pairwise relationships based on raw spectral sequences, 2D-GCN constructs the graph based on GADF-derived image features, thereby allowing the model to exploit inter-band structural relationships encoded in the two-dimensional representation. The same two-layer GCN architecture and training strategy were used for 2D-GCN to ensure a fair comparison with 1D-GCN.

### 2.5. Construction of HGNNs

HGNNs are deep learning frameworks designed to model complex high-order relationships. Unlike ordinary graphs, in which an edge connects only two nodes, a hyperedge in a hypergraph can connect multiple nodes simultaneously, making it more suitable for characterizing potential high-order associations among samples [21,22,23]. In the hyperspectral identification task of *Fritillaria* species, each sample contains its own spectral response information and may also exhibit similarity relationships with other samples in either the spectral feature space or the image-structural feature space. Therefore, a sample-level HGNN model was constructed in this study, in which each *Fritillaria* slice sample was defined as a node.

Let the sample node set be defined as:

(5) $$V=\left\{v_{1},\;v_{2},\;\ldots ,\;v_{N}\right\}\;,$$

where N denotes the number of samples, and $v_{i}$ denotes the i-th *Fritillaria* hyperspectral sample. The initial node attribute matrix is defined as:

(6) $$X^{\left(0\right)}={\left[x_{1},\;x_{2},\;\ldots ,\;x_{N}\right]}^{T}\in R^{N\times d}\;,$$

where d denotes the dimensionality of node features. In an HGNN, node attributes represent the information to be propagated and updated, whereas hyperedges determine which samples can exchange information. Therefore, the key issue in hypergraph modeling is not only to construct a sample relationship structure, but also to clarify two aspects: what features are assigned to the nodes and what features are used to construct hyperedges among samples. Based on this idea, this study constructed HGNN models based on 1D spectra, HGNN models based on 2D GADF images, and the proposed SSD-HGNN model that integrates 1D spectral features and 2D GADF image features.

#### 2.5.1. Construction of HGNN Based on 1D Spectra

First, a sample-level hypergraph model was constructed based on 1D spectra. Let the raw spectrum of the i-th sample be defined as:

(7) $$s_{i}=\left[s_{i1},\;s_{i2},\;\ldots ,\;s_{iB}\right]\;,$$

where B denotes the number of spectral bands. The most direct strategy is to use the raw spectrum as the node attribute:

(8) $$x_{i}^{raw}=s_{i}\;.$$

The raw spectral node attribute matrix of all samples can be expressed as:

(9) $$X_{raw}^{\left(0\right)}={\left[x_{1}^{raw},\;x_{2}^{raw},\;\ldots ,x_{N}^{raw}\right]}^{T}\;.$$

In this case, the HGNN propagates raw reflectance information, and high-order information fusion among different samples is performed through the hypergraph structure. However, raw spectra are shallow features whose representation mainly comes from band-wise reflectance values and may be affected by noise, local fluctuations, and individual sample variations. To enhance the discriminative representation of each node, a 1D-ResNet was further introduced to encode deep features from the raw spectra:

(10) $$h_{i}^{1D}=f_{1D-ResNet}\left(s_{i}\right)\;,$$

where $f_{1D-ResNet}\left(\cdot \right)$ denotes the 1D-ResNet feature extractor, and $h_{i}^{1D}$ denotes the deep spectral feature of the i-th sample. Accordingly, the ResNet-based node attribute matrix is expressed as:

(11) $$X_{res}^{\left(0\right)}={\left[h_{1}^{1D},\;h_{2}^{1D},\;\ldots ,h_{N}^{1D}\right]}^{T}\;.$$

At this stage, the HGNN no longer propagates raw reflectance vectors, but instead propagates deep discriminative features extracted by 1D-ResNet. In other words, 1D-ResNet enhances the feature representation of each node, while the HGNN further integrates information from different nodes based on the sample relationship structure.

A KNN strategy was used to construct sample-level hyperedges. For all HGNN and SSD-HGNN models, K was also set to 5 to ensure consistency with the KNN-based graph construction and to allow fair comparison among different graph- and hypergraph-based models. For the i-th node, its similarity to other samples was first calculated in a specified feature space, and its K nearest neighboring samples were selected:

(12) $$N_{K}\left(v_{i}\right)=\left\{v_{i_{1}},\;v_{i_{2}},\;\ldots ,v_{i_{K}}\right\}\;.$$

The central node and its neighboring nodes were then grouped to form one hyperedge:

(13) $$e_{i}=\left\{v_{i}\right\}\cup N_{K}\left(v_{i}\right)\;.$$

For the 1D spectral hypergraph, two types of hyperedge construction strategies were designed. The first strategy constructed hyperedges based on raw spectra:

(14) $$G=G\left(X_{raw}\right)\;,$$

where sample-level high-order connections were determined directly according to similarities among raw spectra. The second strategy constructed hyperedges based on deep features extracted by 1D-ResNet:

(15) $$G=G\left(X_{res}\right)\;,$$

where high-order relationships among samples were constructed according to similarities among deep spectral features. Compared with raw spectra, 1D-ResNet features generally provide stronger category-discriminative information; therefore, hyperedges constructed from deep features are expected to produce a sample topology that better reflects species-level differences.

Based on different combinations of node attributes and hyperedge construction features, four 1D-HGNN models were constructed.

In the first model, both node attributes and hyperedge construction were based on raw spectra:

(16) $$X^{\left(0\right)}=X_{raw},\;\;G=G\left(X_{raw}\right)\;.$$

This model was denoted as RawSpec-RawSpec-HGNN, where the first “RawSpec” indicates that the node attributes were derived from raw spectra, and the second “RawSpec” indicates that the hyperedges were constructed based on raw spectral similarity. This model served as the most basic 1D hypergraph baseline and was used to evaluate the effect of direct hypergraph propagation based on raw spectra.

In the second model, deep features extracted by 1D-ResNet were used as node attributes, whereas the hyperedges were still constructed from raw spectra:

(17) $$X^{\left(0\right)}=X_{res},\;\;G=G\left(X_{raw}\right)\;.$$

This model was denoted as ResSpec-RawSpec-HGNN, where “ResSpec” indicates that the node attributes were derived from deep spectral features extracted by 1D-ResNet, and “RawSpec” indicates that the hyperedges were constructed based on raw spectral similarity. This model was used to examine whether propagating deep spectral features is more effective than propagating raw spectra under the same raw-spectral topology.

(18) $$X^{\left(0\right)}=X_{raw},\;\;G=G\left(X_{res}\right)\;.$$

This model was denoted as RawSpec-ResSpec-HGNN, in which the propagated node information was derived from raw spectra, while the hypergraph topology was induced by deep spectral features. This model was used to investigate whether deep spectral features can construct more effective high-order sample relationships.

In the fourth model, both node attributes and hyperedge construction were based on deep 1D-ResNet features:

(19) $$X^{\left(0\right)}=X_{res},\;\;G=G\left(X_{res}\right)\;.$$

**Figure 5:**
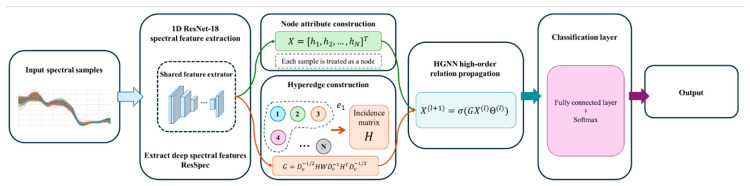
Framework of the ResSpec-ResSpec-HGNN model.

This model was denoted as ResSpec-ResSpec-HGNN and can be regarded as a deep spectral feature-induced 1D-HGNN. It uses 1D-ResNet to enhance node representation and uses deep spectral features to construct a more discriminative hypergraph topology, as shown in Figure 5.

To transform the hyperedge structure into a matrix form suitable for neural network propagation, the node-hyperedge incidence matrix was constructed as:

(20) $$H\in R^{N\times E}\;,$$

where E denotes the number of hyperedges. If node $v_{i}$ belongs to hyperedge $e_{j}$, then:

(21) $$H\left(v_{i},\;e_{j}\right)=1\;,$$

otherwise:

(22) $$H\left(v_{i},\;e_{j}\right)=0\;.$$

Based on this incidence matrix, the hyperedge weight matrix W, node degree matrix $D_{v}$, and hyperedge degree matrix $D_{e}$ were defined. The node degree indicates how many hyperedges a node participates in, whereas the hyperedge degree indicates how many nodes are connected by a hyperedge. The normalized hypergraph propagation matrix is defined as:

(23) $$G=D_{v}^{-\frac{1}{2}}HWD_{e}^{-1}H^{T}D_{v}^{-\frac{1}{2}}\;.$$

This propagation matrix jointly considers the connection relationships between nodes and hyperedges, the frequency with which nodes participate in hyperedges, and the number of nodes contained in each hyperedge, thereby avoiding imbalanced propagation weights caused by differences in node or hyperedge connectivity.

The feature propagation process of the HGNN can be expressed as:

(24) $$X^{\left(l+1\right)}=\sigma \left(GX^{\left(l\right)}\Theta^{\left(l\right)}\right)\;,$$

where $X^{\left(l\right)}$ denotes the node features at the l-th layer, $\Theta^{\left(l\right)}$ denotes the trainable parameter matrix, and $\sigma \left(\cdot \right)$ denotes the nonlinear activation function. For the i-th node, the update process can be expanded as:

(25) $$x_{i}^{\left(l+1\right)}=\sigma \left(\sum_{j=1}^{N}G_{ij}x_{j}^{\left(l\right)}\Theta^{\left(l\right)}\right)\;.$$

This formulation indicates that the new representation of node $v_{i}$ is derived not only from its own features but also from other sample nodes associated with it through hyperedges. Through hypergraph propagation, the HGNN enables each sample to aggregate information from structurally similar samples, thereby enhancing the consistency of intra-class representations and improving the discriminative stability of samples near classification boundaries.

#### 2.5.2. Construction of HGNN Based on GADF Images

After constructing the 1D spectral HGNN, the method was extended to the 2D GADF image modality. In the 2D-HGNN, each *Fritillaria* slice sample was defined as a node. Unlike the 1D-HGNN, both node attributes and hyperedge construction features were derived from GADF image representations. To systematically analyze the role of GADF image features in hypergraph propagation, two types of node features were considered.

The first type is the raw flattened GADF image feature. As HGNN requires a node feature matrix, each GADF image was flattened into a one-dimensional vector:

(26) $$x_{i}^{GADF}=Flatten\left(A_{i}\right)\;,$$

where $A_{i}$ denotes the GADF image of the i-th sample, and $x_{i}^{GADF}$ represents its raw flattened feature. The raw GADF feature matrix of all samples is expressed as:

(27) $$X_{GADF}^{raw}={\left[x_{1}^{GADF},\;x_{2}^{GADF},\;\ldots ,x_{N}^{GADF}\right]}^{T}\;.$$

The second type is the deep GADF image feature extracted by 2D-ResNet. 2D-ResNet18 was trained on the *Fritillaria* GADF image classification task, and the final classification layer was removed to extract deep features:

(28) $$h_{i}^{GADF}=f_{2D-ResNet}\left(A_{i}\right)\;,$$

where $f_{2D-ResNet}\left(\cdot \right)$ denotes the 2D-ResNet18 feature extractor, and $h_{i}^{GADF}$ denotes the deep GADF feature of the i-th sample. The deep feature matrix of all samples is:

(29) $$H_{GADF}^{res}={\left[h_{1}^{GADF},\;h_{2}^{GADF},\;\ldots ,h_{N}^{GADF}\right]}^{T}\;.$$

Compared with raw flattened features, deep GADF features contain higher-level structural information and class-discriminative information due to supervised training.

**Figure 6:**
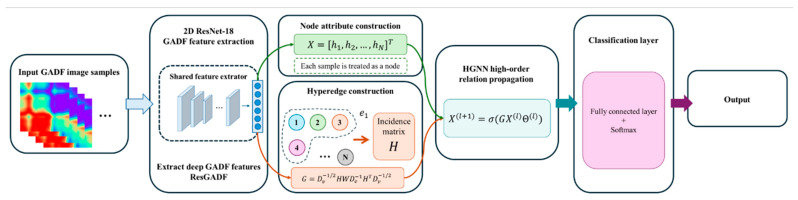
Framework of the ResGADF-ResGADF-HGNN model.

Based on these node features, four 2D-HGNN models were constructed in a manner consistent with Section 2.5.1. The first model, with both node attributes and hyperedges based on raw flattened GADF features, was denoted as RawGADF-RawGADF-HGNN. The second model used GADF-ResNet deep features as node attributes while hyperedges were constructed from raw flattened GADF features, denoted as ResGADF-RawGADF-HGNN. The third model used raw flattened GADF features as node attributes while hyperedges were constructed from GADF-ResNet deep features, denoted as RawGADF-ResGADF-HGNN. The fourth model used GADF-ResNet deep features for both node attributes and hyperedges, denoted as ResGADF-ResGADF-HGNN, as shown in Figure 6. All four models adopted the same hyperedge construction and propagation mechanism as in Section 2.5.1.

#### 2.5.3. Proposed SSD-HGNN Model

Based on the 1D spectral HGNN and the 2D GADF HGNN, this study further proposed a SSD-HGNN that integrates 1D spectral features and 2D GADF image features. Unlike simple concatenation of 1D spectral features and 2D image features, SSD-HGNN adopts a decoupled fusion strategy between node attributes and hyperedge construction features, allowing the two modalities to play different roles in hypergraph learning. Specifically, deep 1D spectral features are used as node attributes to provide discriminative information to be propagated, whereas deep 2D GADF image features are used for hyperedge construction to determine how high-order information is exchanged among samples.

Specifically, 1D-ResNet was first used to extract deep features from the raw spectra, generating the deep 1D spectral feature of the i-th sample:

(30) $$h_{i}^{1D}=f_{1D-ResNet}\left(s_{i}\right)\;,$$

where $s_{i}$ denotes the raw 1D spectrum of the i-th sample, $f_{1D-ResNet}\left(\cdot \right)$ denotes the 1D-ResNet feature extractor, and $h_{i}^{1D}$ denotes the corresponding deep spectral feature. The 1D ResNet feature matrix of all samples is expressed as:

(31) $$H_{1D}^{res}={\left[h_{1}^{1D},\;h_{2}^{1D},\;\ldots ,h_{N}^{1D}\right]}^{T}\;.$$

This feature matrix was used as the initial node attribute of SSD-HGNN:

(32) $$X^{\left(0\right)}=H_{1D}^{res}\;.$$

Therefore, in SSD-HGNN, the information propagated and updated through the hypergraph is the deep spectral feature extracted by 1D-ResNet. This design preserves the direct discriminative information embedded in the original spectral sequence and provides each node with strong spectral representation ability.

Meanwhile, 2D-ResNet was used to extract deep features from the GADF images, generating the deep GADF image feature of the i-th sample:

(33) $$h_{i}^{GADF}=f_{2D-ResNet}\left(A_{i}\right)\;,$$

where $A_{i}$ denotes the GADF image corresponding to the i-th sample, $f_{2D-ResNet}\left(\cdot \right)$ denotes the 2D-ResNet feature extractor, and $h_{i}^{GADF}$ denotes the corresponding deep GADF image feature. The GADF-ResNet feature matrix of all samples is expressed as:

(34) $$H_{GADF}^{res}={\left[h_{1}^{GADF},\;h_{2}^{GADF},\;\ldots ,h_{N}^{GADF}\right]}^{T}\;.$$

Unlike the node attributes, $H_{GADF}^{res}$ was not directly used as the information to be propagated in the HGNN. Instead, it was used to construct sample-level hyperedges:

(35) $$G=G\left(H_{GADF}^{res}\right)\;.$$

That is, the K nearest neighbors of each sample were searched in the GADF-ResNet deep feature space, and each central sample and its neighboring samples were grouped to form a hyperedge. The resulting hypergraph structure reflects high-order similarity relationships among samples in the 2D GADF structural feature space.

Therefore, the proposed SSD-HGNN can be expressed as:

(36) $$X^{\left(0\right)}=H_{1D}^{res},\;\;G=G\left(H_{GADF}^{res}\right)\;,$$

and feature propagation is performed through hypergraph convolution:

(37) $$X^{\left(l+1\right)}=\sigma \left(GX^{\left(l\right)}\Theta^{\left(l\right)}\right)\;,$$

**Figure 7:**
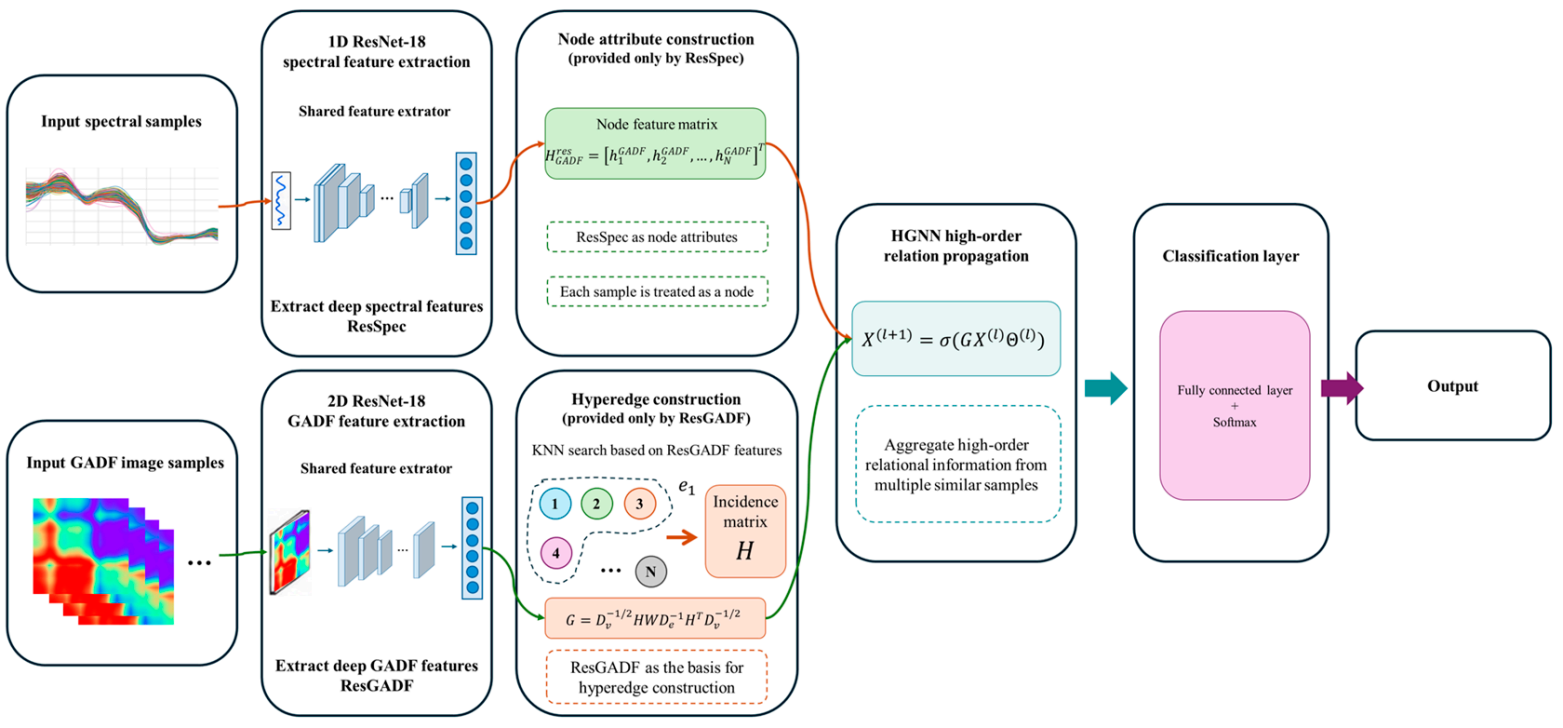
Framework of the proposed SSD-HGNN model.

In this model, the 1D spectral modality provides the “information to be propagated”, whereas the 2D GADF image modality provides the “rules for information propagation”. This attribute-topology decoupled fusion strategy avoids the potential redundancy caused by simple feature concatenation and allows the model to jointly exploit the discriminative features of 1D spectral sequences and the inter-band structural relationships of 2D GADF images. Compared with pure 1D-HGNN, SSD-HGNN introduces the 2D inter-spectral structural information represented by GADF images during hyperedge construction. Compared with pure 2D-HGNN, SSD-HGNN retains the direct discriminative representation of the original spectral sequence through 1D spectral ResNet features during node propagation. Therefore, SSD-HGNN enables collaborative modeling of 1D spectral information and 2D GADF structural information in a sample-level hypergraph, providing a more comprehensive multimodal hypergraph representation for the species identification of ZBM and HBBM, as shown in Figure 7.

The same reference-set-based hypergraph inference strategy was used for all HGNN and SSD-HGNN models. During validation and test inference, validation or test samples were inserted into the corresponding hypergraph as unlabeled nodes together with the training samples. Their labels were strictly masked and were not used for hyperedge construction, incidence matrix calculation, loss calculation, or parameter optimization. Hyperedges were constructed only based on feature similarity, such as raw spectral features, ResNet-extracted spectral features, raw GADF features, or ResNet-extracted GADF features. Therefore, the validation and test samples provided only unlabeled feature information for establishing sample relationships, and no label information was introduced into model training. This strategy is consistent with practical quality-control applications, where a new unknown *Fritillaria* sample can be inserted into an existing reference hypergraph and connected to labeled reference samples according to feature similarity for species inference.

### 2.6. Post-Hoc Interpretability Analysis

Although explainability has been extensively investigated for conventional graph neural networks, dedicated interpretability methods for hypergraph neural networks remain relatively limited. A recent review indicated that HGNN explainability is still at an early stage and highlighted the identification of influential nodes, hyperedges, and sub-hypergraphs, as well as the characterization of information interaction and aggregation, as important research directions [39]. Existing studies have mainly explored intrinsic attention or learnable weighting mechanisms and post-hoc explanatory sub-hypergraph extraction. For example, Bai et al. [40] introduced hypergraph attention to dynamically weight node–hyperedge interactions, whereas HyperEX assigns importance scores to node–hyperedge incidences and extracts influential sub-hypergraphs as local explanations [41]. More recently, SHypX searched for concise sub-hypergraphs that preserve the original model predictions to provide local and global explanations [42]. Nevertheless, these approaches remain limited in number and often depend on particular model structures or explanation objectives.

Considering that SSD-HGNN does not contain an explicit attention mechanism and uses a fixed inductive hypergraph structure, a perturbation-based post-hoc sensitivity analysis was conducted without modifying or retraining the original model. Specifically, spectral-region occlusion was used to identify the wavelength intervals to which the model predictions were most sensitive, whereas individual hyperedge removal was used to quantify the influence of the structural relationship associated with each test sample. For spectral-region occlusion, a sliding window containing 10 consecutive bands was replaced with the corresponding band-wise mean calculated from the training set. The occluded spectra were subsequently passed through the one-dimensional spectral feature extractor, while the original hypergraph structure was retained to isolate the sensitivity of the spectral attribute branch. Spectral-region importance was quantified by the decrease in the predicted probability of the true class and the corresponding decrease in test accuracy relative to the unoccluded input.

For hyperedge analysis, the inductive hyperedge associated with each test sample was individually removed from the incidence matrix, while all other model components and inputs remained unchanged. Its importance was quantified as:

(38) $$I\left(e\right)=p_{y}\left(X,\;H\right)-p_{y}(X,\;H\;\backslash \;e)\;,$$

where $p_{y}\left(X,\;H\right)$ denotes the predicted probability assigned to the true class under the original hypergraph, and $p_{y}(X,\;H\;\backslash \;e)$ denotes the corresponding probability after removing hyperedge e. A larger positive value of $I\left(e\right)$ indicates that removing the corresponding hyperedge produces a greater reduction in the true-class probability and, therefore, that the prediction is more sensitive to this hyperedge. These importance scores represent post-hoc model sensitivity rather than direct causal evidence.

### 2.7. Model Evaluation and Software

The classification task in this study was a binary identification problem between ZBM and HBBM, and the numbers of samples in the two classes were relatively balanced across the training, validation, and test sets. Therefore, classification accuracy was used as the primary evaluation metric to measure the overall discriminative performance of different models. In addition, to provide a more comprehensive evaluation of model performance, precision, recall, and F1-score were also calculated. Accuracy, precision, recall, and F1-score were calculated as follows:

(39) $$Accuracy=\frac{\left(TP+TN\right)}{\left(TP+TN+FP+FN\right)}\;,$$

(40) $$Precision=\frac{TP}{\left(TP+FP\right)}\;,$$

(41) $$Recall=\frac{TP}{\left(TP+FN\right)}\;,$$

(42) $$F1-score=\frac{2\times Precision\times Recall}{\left(Precision+Recall\right)}\;,$$

where TP denotes the number of true positive samples, TN denotes the number of true negative samples, FP denotes the number of false positive samples, and FN denotes the number of false negative samples. Accuracy reflects the overall proportion of correctly classified samples. Precision measures the proportion of correctly predicted positive samples among all samples predicted as positive. Recall reflects the proportion of correctly predicted positive samples among all actual positive samples. F1-score is the harmonic mean of precision and recall and provides a balanced evaluation of classification performance. To systematically assess model fitting, model selection, and generalization performance, the above evaluation metrics were calculated on the training, validation, and test sets, respectively. The training-set results were used to evaluate model fitting behavior, the validation-set results were used for model selection and hyperparameter adjustment, and the test-set results were used for final performance evaluation [43].

All data analyses were conducted on a Windows 11 64-bit operating system with an Intel Core i7-14700KF CPU and an NVIDIA GeForce RTX 4070 Ti SUPER 16GB GPU. Algorithm development and execution were performed using PyCharm 2024.1 and Python 3.12.9. The machine learning models, including LR and SVC, were implemented using Scikit-learn 1.6.0, and XGBoost was implemented using the XGBoost library 3.2.0. The deep learning models, including CNN, Transformer, CNN-Transformer, ResNet, GCN, HGNN, and SSD-HGNN, were constructed using PyTorch 2.5.1. All deep learning models were executed on the GPU.

## 3. Results

### 3.1. Spectral Characteristics

**Figure 8:**
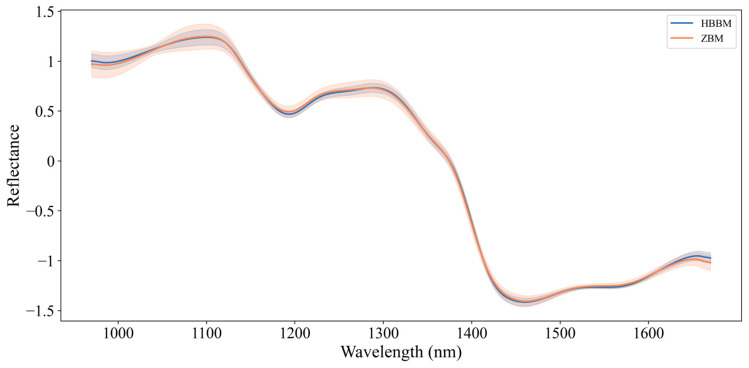
Mean spectra and wavelength-wise standard deviations of ZBM and HBBM after SNV preprocessing.

In this study, the raw spectra were first smoothed using a moving average method with a five-point window, followed by SNV preprocessing to reduce the effects of scattering and baseline drift. Figure 8 shows the mean spectra of ZBM and HBBM after smoothing and SNV preprocessing, together with the standard deviation at each wavelength. As shown in Figure 8, the preprocessed reflectance spectra of ZBM and HBBM exhibited noticeable differences. These differences were more pronounced at the beginning and end of the spectral range, as well as near the reflectance peaks and valleys, indicating that ZBM and HBBM possessed distinguishable spectral characteristics.

### 3.2. Results of Models Constructed Using 1D Spectra

In this study, classification models were first constructed based on the preprocessed 1D spectra. The samples of ZBM and HBBM were divided into training, validation, and test sets at a ratio of 4:1:1, and the detailed sample numbers are listed in Table 1. Table 2 presents the classification performance of different models constructed using 1D spectra on the training, validation, and test sets. Accuracy, precision, recall, and F1-score were calculated to comprehensively evaluate the classification performance of each model. Overall, all models achieved effective discrimination between ZBM and HBBM, indicating that the preprocessed 1D spectra contained discriminative information related to species differences. However, clear performance differences were observed among different modeling strategies.

Among the conventional machine learning models, SVC achieved the best performance, with accuracies of 0.9705, 0.9457, and 0.9408 on the training, validation, and test sets, respectively. Its test precision, recall, and F1-score were 0.9417, 0.9396, and 0.9404, respectively, which were consistent with its high test accuracy. LR achieved a test accuracy of 0.9093 and a test F1-score of 0.9088, suggesting that the linear model could capture part of the spectral differences between ZBM and HBBM, although its representational capacity was limited. XGBoost achieved a training accuracy of 0.9438, but its validation and test accuracies decreased to 0.8887 and 0.8740, respectively. Its test precision, recall, and F1-score were also lower than those of LR and SVC, indicating relatively insufficient generalization ability. Overall, conventional machine learning models were able to exploit the basic discriminative information contained in the 1D spectra, but their ability to capture complex nonlinear spectral patterns and deep features remained limited.

Among the conventional deep learning models, 1D-CNN, 1D-ResNet18, and 1D-ResNet34 all achieved high classification performance. 1D-CNN achieved accuracies of 0.9580, 0.9435, and 0.9479 on the training, validation, and test sets, respectively, and obtained the highest test accuracy among all 1D spectral models. Its test precision, recall, and F1-score were 0.9483, 0.9470, and 0.9476, respectively, indicating stable classification performance across different evaluation metrics. This result demonstrates that the one-dimensional convolutional structure could effectively extract local response features between adjacent wavelengths. 1D-ResNet18 achieved accuracies of 0.9810, 0.9463, and 0.9452 on the training, validation, and test sets, respectively, showing strong performance on both validation and test sets. In contrast, 1D-ResNet34 obtained a test accuracy of 0.9441, which was slightly lower than that of 1D-ResNet18. This suggests that, for the dataset used in this study, a residual network with moderate depth was sufficient to extract discriminative spectral features, whereas further increasing the network depth did not lead to additional improvement.

The Transformer and CNN-Transformer models were further constructed to evaluate the performance of attention-based spectral modeling. The Transformer model achieved accuracies of 0.9274, 0.9180, and 0.9159 on the training, validation, and test sets, respectively, with a test precision, recall, and F1-score of 0.9177, 0.9138, and 0.9152, respectively. Compared with Transformer, CNN-Transformer achieved better performance, with accuracies of 0.9511, 0.9414, and 0.9349 on the training, validation, and test sets, respectively. Its test precision, recall, and F1-score were 0.9350, 0.9341, and 0.9345, respectively. These results indicate that introducing convolutional layers before Transformer encoding was beneficial for extracting local spectral features and improving classification performance. However, both Transformer-related models showed lower test accuracy than 1D-CNN and 1D-ResNet18, suggesting that convolutional and residual structures were more effective for this ZBM and HBBM binary classification task under the current dataset and model settings.

After introducing graph-structured modeling, 1D-GCN achieved accuracies of 0.8938, 0.8931, and 0.8789 on the training, validation, and test sets, respectively. Its test precision, recall, and F1-score were 0.8790, 0.8789, and 0.8788, respectively. Compared with 1D-CNN and 1D-ResNet18, the performance of 1D-GCN was clearly lower, indicating that pairwise relationship propagation based solely on a conventional graph structure did not effectively improve classification performance. Although the KNN graph can connect each sample to multiple nearest neighbors, these connections are essentially multiple independent pairwise edges and are therefore insufficient for characterizing the overall high-order relationships among multiple similar samples.

Compared with 1D-GCN, the HGNN models based on one-dimensional spectra showed better overall performance. RawSpec-RawSpec-HGNN achieved a test accuracy of 0.9186 and a test F1-score of 0.9183, which were higher than those of 1D-GCN. This indicates that the hypergraph structure was more effective than the conventional graph structure in modeling relationships among spectral samples. After further introducing deep features extracted by 1D-ResNet18, the model performance improved substantially. ResSpec-RawSpec-HGNN achieved accuracies of 0.9893, 0.9533, and 0.9414 on the training, validation, and test sets, respectively, and its test precision, recall, and F1-score were 0.9424, 0.9401, and 0.9410, respectively. These results demonstrate that using deep ResNet features as node attributes enhanced the discriminative ability of the features propagated in HGNN. The test accuracy of RawSpec-ResSpec-HGNN was 0.8947, which was lower than those of the other one-dimensional HGNN models, suggesting that when only the hyperedge construction features were optimized while the propagated node attributes remained raw spectral features, the model performance was still constrained by the limited representational capacity of the raw node features.

Among the four 1D HGNN models, ResSpec-ResSpec-HGNN achieved the best test performance, with accuracies of 0.9890, 0.9533, and 0.9425 on the training, validation, and test sets, respectively. Its test precision, recall, and F1-score were 0.9433, 0.9413, and 0.9421, respectively. In this model, deep spectral features extracted by 1D-ResNet18 were used simultaneously as node attributes and as the basis for hyperedge construction, enabling HGNN to perform high-order relationship propagation in a more discriminative feature space. On the one hand, the propagated information consisted of spectral features encoded by the deep network; on the other hand, the hyperedges were also determined by sample similarities in the deep feature space, thereby forming high-order sample connections that better matched the class distribution.

In summary, the results in Table 2 show that SVC was the best-performing conventional machine learning model, while 1D-CNN and 1D-ResNet18 achieved the strongest performance among the conventional deep learning models. Transformer and CNN-Transformer also achieved effective classification performance, but they did not outperform the convolutional and residual models in this study. The relatively poor performance of 1D-GCN indicates that pairwise graph-based propagation was insufficient for modeling complex spectral sample relationships. In contrast, HGNN could connect multiple similar samples through hyperedges and further exploit high-order relationships among samples. Among the one-dimensional HGNN variants, ResSpec-ResSpec-HGNN achieved the best test performance, indicating that the combination of deep spectral feature extraction and sample-level high-order relationship modeling contributed to improved species recognition of ZBM and HBBM. In addition, the trends of precision, recall, and F1-score were generally consistent with those of accuracy across different models, suggesting that the classification performance was relatively stable across multiple evaluation metrics.

### 3.3. Results of Models Constructed Using GADF Images

Table 3 presents the classification results of different models constructed using GADF images on the training, validation, and test sets. Accuracy, precision, recall, and F1-score were used to comprehensively evaluate model performance. Overall, after converting the one-dimensional spectra into two-dimensional GADF images, all models were able to discriminate between ZBM and HBBM effectively. This indicates that GADF images preserved the species-related discriminative information contained in the original spectra and represented the relative variation relationships among different wavelength bands in a two-dimensional structural form.

Among the conventional two-dimensional deep learning models, 2D-CNN, 2D-ResNet18, and 2D-ResNet34 all used GADF images as model inputs for classification. The 2D-CNN model achieved accuracies of 0.9606, 0.9381, and 0.9229 on the training, validation, and test sets, respectively. Its test precision, recall, and F1-score were 0.9346, 0.9334, and 0.9339, respectively, indicating that the two-dimensional convolutional structure could extract useful local texture and spatial structural features from GADF images. The 2D-ResNet18 model achieved accuracies of 0.9868, 0.9332, and 0.9321 on the training, validation, and test sets, respectively, with a test precision, recall, and F1-score of 0.9345, 0.9300, and 0.9315, respectively. Compared with 2D-CNN, 2D-ResNet18 achieved higher test accuracy, suggesting that residual learning was beneficial for enhancing feature extraction from GADF images. The 2D-ResNet34 model reached a training accuracy of 0.9947, whereas its validation and test accuracies were 0.9273 and 0.9316, respectively. Its test precision, recall, and F1-score were 0.9318, 0.9307, and 0.9312, respectively. Further increasing the network depth from 2D-ResNet18 to 2D-ResNet34 did not lead to additional improvement, which may be related to the training sample size, redundancy in image features, and the risk of overfitting.

After further introducing graph-structured modeling, 2D-GCN achieved accuracies of 0.9362, 0.8979, and 0.8893 on the training, validation, and test sets, respectively. Its test precision, recall, and F1-score were 0.8892, 0.8882, and 0.8886, respectively. Compared with 2D-CNN and 2D-ResNet, 2D-GCN showed clearly lower classification performance. This indicates that directly constructing a conventional KNN graph based on GADF features and performing GCN propagation did not effectively improve the classification performance of the two-dimensional GADF models. 2D-GCN mainly models pairwise relationships among samples and is therefore limited in capturing high-order associations among multiple similar samples.

Compared with 2D-GCN, the HGNN models based on GADF images achieved better overall performance. RawGADF-RawGADF-HGNN directly used raw GADF image features as both node attributes and the basis for hyperedge construction, achieving accuracies of 0.9856, 0.9327, and 0.9224 on the training, validation, and test sets, respectively. Its test precision, recall, and F1-score were 0.9219, 0.9222, and 0.9221, respectively. Compared with 2D-GCN, its test accuracy increased from 0.8893 to 0.9224, suggesting that the hypergraph structure could model high-order relationships among GADF samples more effectively than the conventional graph structure.

After introducing deep GADF image features extracted by ResNet, the classification performance of the two-dimensional HGNN models was further improved. ResGADF-RawGADF-HGNN used ResNet-extracted deep GADF features as node attributes and raw GADF image features for hyperedge construction. It achieved accuracies of 0.9940, 0.9349, and 0.9273 on the training, validation, and test sets, respectively. Its test precision, recall, and F1-score were 0.9278, 0.9261, and 0.9268, respectively. Compared with RawGADF-RawGADF-HGNN, this model improved both validation and test performance, indicating that using deep GADF features extracted by ResNet as node attributes could enhance the discriminative ability of the information propagated in HGNN.

RawGADF-ResGADF-HGNN used raw GADF image features as node attributes and ResNet-extracted deep GADF features for hyperedge construction. It achieved accuracies of 1.0000, 0.9349, and 0.9245 on the training, validation, and test sets, respectively. Its test precision, recall, and F1-score were 0.9243, 0.9241, and 0.9242, respectively. Although the training accuracy reached 1.0000, the validation and test accuracies were clearly lower than the training accuracy, indicating potential overfitting. This phenomenon may be related to the high-dimensional raw GADF node attributes and the increased model complexity introduced by hypergraph construction. These results suggest that when only the hyperedge construction features were optimized using deep GADF features while the propagated node attributes remained raw flattened GADF features, model performance could still be constrained by the limited generalization ability of the raw node representation.

ResGADF-ResGADF-HGNN simultaneously used ResNet-extracted deep GADF features as both node attributes and the basis for hyperedge construction. It achieved accuracies of 0.9950, 0.9349, and 0.9251 on the training, validation, and test sets, respectively. Its test precision, recall, and F1-score were 0.9255, 0.9241, and 0.9246, respectively. Although its test accuracy was slightly lower than that of ResGADF-RawGADF-HGNN, both its node attributes and hyperedge construction basis were derived from supervised ResNet-extracted deep GADF features. Therefore, this model provided a structurally consistent way to enhance both node representation and hypergraph topology construction in the GADF feature space.

Taken together, the results of the two-dimensional single-modality models in Table 3 demonstrate that GADF images can provide effective two-dimensional structural information for ZBM and HBBM recognition. The 2D-CNN and 2D-ResNet models were able to extract local texture and spatial structural features from GADF images, while the HGNN models further exploited high-order relationships among GADF samples through hyperedges. Among the conventional GADF-based deep learning models, 2D-ResNet18 achieved the highest test accuracy. Among the GADF-based HGNN models, ResGADF-RawGADF-HGNN achieved the highest test accuracy. In addition, the trends of precision, recall, and F1-score were generally consistent with those of accuracy, indicating that the classification results were relatively stable across different evaluation metrics. However, compared with the 1D spectral models, the 2D GADF-based models did not show clear overall superiority. Therefore, GADF images should be regarded as a complementary structural representation rather than a direct replacement for one-dimensional spectra. Their main value in this study lies in providing additional inter-band structural information for subsequent spectral–structural decoupled hypergraph fusion.

### 3.4. Results of the Proposed SSD-HGNN Model

On the basis of the 1D spectral models and 2D GADF image models, this study further constructed SSD-HGNN to integrate one-dimensional spectral information with two-dimensional GADF structural information. Unlike direct feature concatenation of one-dimensional and two-dimensional representations, SSD-HGNN adopts a decoupled strategy between node attributes and hyperedge construction. Specifically, the deep spectral features extracted by 1D-ResNet18 are used as node attributes, whereas the deep GADF image features extracted by 2D-ResNet18 are used as the basis for hyperedge construction. In other words, the one-dimensional spectral modality mainly provides the node-level discriminative information to be propagated, whereas the two-dimensional GADF modality mainly provides the high-order connection relationships among samples, thereby achieving a functional division between spectral representation and structural topology.

As shown in Table 3, SSD-HGNN achieved accuracies of 0.9848, 0.9533, and 0.9441 on the training, validation, and test sets, respectively. Its precision, recall, and F1-score were 0.9847, 0.9847, and 0.9847 on the training set; 0.9528, 0.9535, and 0.9531 on the validation set; and 0.9438, 0.9439, and 0.9438 on the test set, respectively. These results show that SSD-HGNN maintained stable classification performance across different evaluation metrics, and its precision, recall, and F1-score were generally consistent with its accuracy. Compared with ResSpec-ResSpec-HGNN, which achieved the best test performance among the one-dimensional spectral HGNN models, SSD-HGNN achieved the same validation accuracy of 0.9533 and improved the test accuracy from 0.9425 to 0.9441. The test F1-score also increased from 0.9421 to 0.9438. Although this improvement was modest, it suggests that introducing two-dimensional GADF-derived structural information for hyperedge construction can provide complementary information to one-dimensional spectral node attributes. Compared with the GADF-based HGNN models, SSD-HGNN showed a more evident improvement. For example, ResGADF-RawGADF-HGNN achieved the highest test accuracy among the GADF-based HGNN variants, with a test accuracy of 0.9273 and a test F1-score of 0.9268. In contrast, SSD-HGNN increased the test accuracy and F1-score to 0.9441 and 0.9438, respectively. This indicates that using one-dimensional deep spectral features as node attributes while using two-dimensional GADF deep features for hyperedge construction was more effective than relying only on GADF-derived features for both node representation and hypergraph construction.

In addition, the results of SSD-HGNN indicate that effective multimodal fusion does not necessarily require simply stacking all modal features into a high-dimensional input. Instead, assigning different modal features to node representation and topology construction enables the model to exploit their respective advantages within the hypergraph learning framework. Compared with conventional deep learning models, SSD-HGNN not only learns deep features from individual samples, but also models high-order relationships among multiple similar samples through hyperedge propagation. Compared with single-modality HGNN models, SSD-HGNN simultaneously uses one-dimensional spectral sequence features and two-dimensional GADF structural features, allowing the two modalities to complement each other in the hypergraph space.

Taken together, the results in Table 2 and Table 3 show that SSD-HGNN achieved competitive overall performance with a test accuracy of 0.9441 and a test F1-score of 0.9438. Although its test accuracy was slightly lower than those of 1D-CNN and 1D-ResNet18, SSD-HGNN reached the highest validation accuracy level and showed stable performance across accuracy, precision, recall, and F1-score. These results indicate that the proposed SSD-HGNN can effectively integrate spectral discriminative information and structural topological information in hyperspectral data, providing a competitive spectral–structural decoupled hypergraph fusion strategy for the rapid and nondestructive identification of ZBM and HBBM.

### 3.5. Post-Hoc Interpretability Results

**Figure 9:**
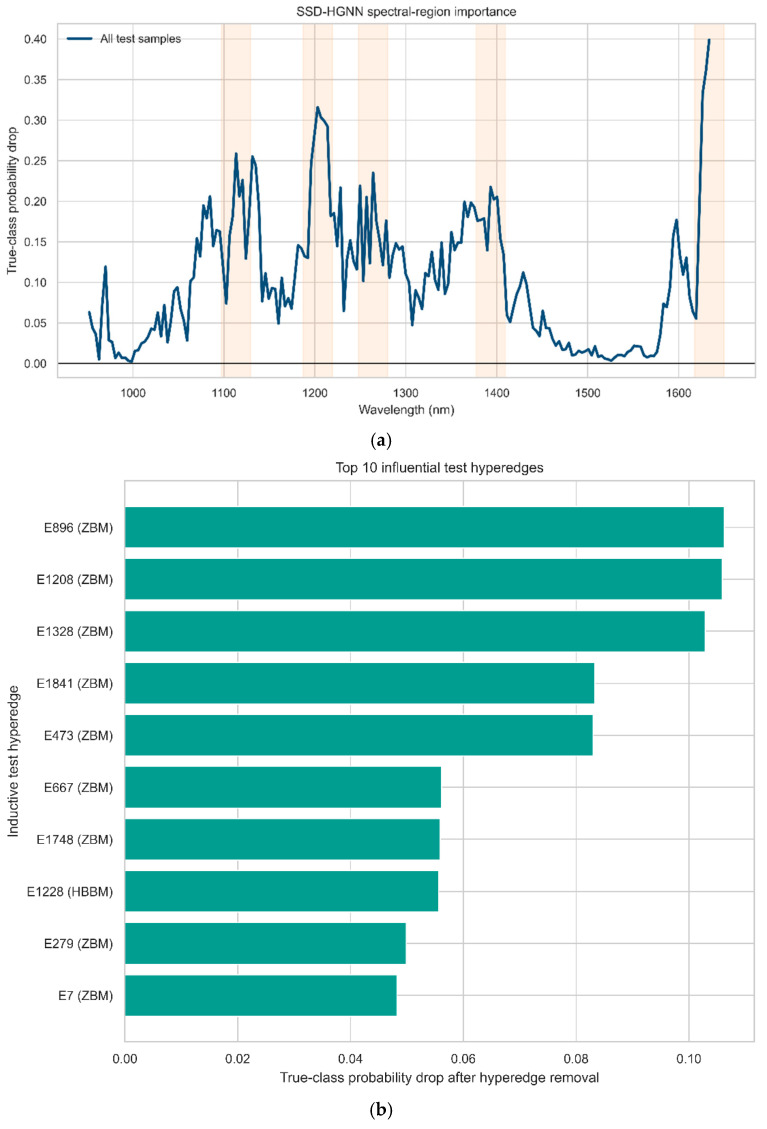
Post-hoc interpretability results of SSD-HGNN. (**a**) Spectral-region importance for all test samples evaluated using sliding-window occlusion. The highlighted regions indicate the five most influential non-overlapping wavelength intervals. (**b**) The ten most influential inductive test hyperedges ranked according to the decrease in the predicted probability of the true class after individual removal.

Spectral-region occlusion revealed that the SSD-HGNN predictions exhibited markedly different sensitivities across the investigated wavelength range, as shown in Figure 9a. Among the evaluated regions, occlusion of the 1617.49–1649.78 nm interval produced the largest change, decreasing the mean predicted probability of the true class by 0.3990 and reducing the test accuracy by 0.4104. Other highly influential intervals included 1187.00–1219.28 nm, 1097.31–1129.60 nm, 1247.98–1280.27 nm, and 1377.13–1409.42 nm. Their corresponding decreases in the mean true-class probability were 0.3159, 0.2588, 0.2351, and 0.2176, respectively. These results demonstrate that the model predictions were more sensitive to several localized wavelength intervals than to the remaining spectral regions.

The hyperedge-removal results are presented in Figure 9b. For 89.25% of the test samples, removal of the associated inductive hyperedge resulted in some reduction in the predicted probability of the true class. However, the magnitude of the change varied substantially among test samples. The three most influential hyperedges were E896, E1208, and E1328, whose individual removal decreased the corresponding true-class probabilities by 0.1063, 0.1059, and 0.1029, respectively. Overall, these results confirm the importance of hypergraph structural information in SSD-HGNN predictions, while also showing that the influence of individual hyperedges varied across test samples.

### 3.6. Computational Cost Analysis

To assess the computational feasibility of the proposed method, the number of model parameters, average inference time per sample, and peak GPU memory consumption were measured for all comparison models. All measurements were performed on an NVIDIA GeForce RTX 4070 Ti SUPER GPU.

As shown in Table 4, the one-dimensional baselines generally exhibited the lowest computational requirements. The 1D-CNN required 2.3081 M parameters, 0.0112 ms per sample, and only 19.6162 MB of peak GPU memory. The Transformer and CNN-Transformer contained relatively few parameters but required 125.0449 and 141.3687 MB of peak memory, respectively, mainly because the self-attention operations retained intermediate representations across the spectral sequence. The 1D-ResNet18 and 1D-ResNet34 also maintained relatively low inference times and memory consumption. Therefore, conventional one-dimensional models remain advantageous when minimum latency and memory consumption are the primary deployment requirements.

The one-dimensional spectral HGNN variants contained 0.3674–4.3720 M parameters and required 74.6455–101.0977 MB of peak GPU memory. Their inference times ranged from 0.2545 to 0.3679 ms per sample. Although these values were higher than those of the conventional one-dimensional models, their absolute inference times remained below 0.4 ms per sample. The additional computational cost was mainly associated with reference-based nearest-neighbor retrieval, inductive hypergraph construction, and hypergraph message propagation.

The two-dimensional models generally required more computational resources because they operated on GADF image representations. In particular, the 2D-GCN required 1452.2979 MB of peak GPU memory and 1.3719 ms per sample. The computational difference among the GADF-based HGNN variants was strongly affected by the type of node attributes. RawGADF-RawGADF-HGNN and RawGADF-ResGADF-HGNN, which directly used flattened raw GADF images as node attributes, required more than 5.7 GB of peak GPU memory. This substantial consumption resulted from propagating high-dimensional raw image vectors during hypergraph learning. By contrast, using compact ResNet-extracted GADF features as node attributes reduced peak GPU memory to approximately 395 MB, as observed for ResGADF-RawGADF-HGNN and ResGADF-ResGADF-HGNN. These results demonstrate that deep feature compression is important for controlling the computational cost of GADF-based graph and hypergraph models.

SSD-HGNN contained 16.5999 M parameters, required 0.3122 ms per sample, and consumed 414.0625 MB of peak GPU memory. Its parameter count was higher than those of the one-dimensional models because both the one-dimensional spectral attribute extractor and the two-dimensional GADF structural extractor were included during inference. Nevertheless, its inference time was lower than those of the 2D-ResNet18, 2D-ResNet34, 2D-GCN, and three of the four GADF-based HGNN variants. Moreover, compared with the two models using raw GADF images as node attributes, SSD-HGNN reduced peak GPU memory consumption by more than 92%, indicating that the use of compact deep features effectively avoids the excessive memory cost of direct raw-image propagation.

SSD-HGNN was therefore not the lightest model in the comparison. The 1D-CNN achieved a slightly higher test accuracy with considerably lower computational cost, whereas SSD-HGNN achieved the highest validation accuracy and competitive test accuracy while explicitly integrating deep spectral node attributes with complementary GADF-based hyperedge topology. Thus, the principal advantage of SSD-HGNN lies in its attribute–topology decoupled representation rather than in achieving the lowest computational cost or the highest test accuracy alone. Overall, the results indicate that SSD-HGNN provides a practical balance among classification performance, spectral–structural representation capability, inference speed, and memory consumption. Further lightweight backbone design, feature dimensionality reduction, and more efficient hypergraph construction will be investigated to improve its suitability for resource-constrained and real-time applications.

## 4. Discussion

ZBM and HBBM slices are difficult to distinguish by visual inspection alone after drying and slicing. In this study, hyperspectral imaging was employed to acquire spectral signals, and classification models were constructed based on spectral features to achieve rapid and nondestructive discrimination between ZBM and HBBM. The quality and commercial value of medicine–food homologous *Fritillaria* materials are affected by species and geographical origin. Previous studies have used near-infrared spectral features to achieve rapid and nondestructive identification of different Fritillaria species [44,45,46,47]. In these studies, multiple Fritillaria species were investigated, and the identification accuracy for different species generally exceeded 90%. Hu et al. [48] applied hyperspectral imaging combined with a CNN algorithm to identify multiple Fritillaria species, including ZBM and HBBM, and achieved favorable results, with discrimination accuracies for ZBM and HBBM exceeding 95%. These findings are generally consistent with the results of the present study, indicating that spectral information contains useful discriminative features for Fritillaria species identification.

The spectral differences between ZBM and HBBM may be related to their differences in chemical composition and biological characteristics. In the near-infrared region used in this study, spectral responses are mainly associated with overtone and combination vibrations of chemical bonds such as O–H, C–H, and N–H, which are closely related to water, carbohydrates, proteins, amino acids, and other organic compounds [49]. *Fritillaria* species contain various characteristic constituents, including steroidal alkaloids, polysaccharides, and flavonoids, and differences in these constituents may contribute to the spectral separability between ZBM and HBBM [50]. Recent studies have also demonstrated that near-infrared spectroscopy combined with deep learning can be effectively used for *Fritillaria* quality control and species identification, further supporting the feasibility of using spectral differences to distinguish *Fritillaria* materials [47].

In this study, modeling analyses were performed using both one-dimensional spectral data and GADF images transformed from the one-dimensional spectra, and both data representations achieved good classification performance. These results demonstrate that both one-dimensional spectra and GADF images can be used for the species identification of ZBM and HBBM. One-dimensional spectra directly describe the relationship between wavelength and spectral response, which facilitates direct spectral modeling and preserves the original sequential information of the spectra. Existing studies on near-infrared spectral identification of *Fritillaria* have mainly used one-dimensional spectral data as model input and have obtained satisfactory results [44,45,46,47]. In the present study, the results of 1D-CNN and 1D-ResNet18 further indicate that convolutional and residual structures can effectively extract discriminative features from one-dimensional spectra. In particular, 1D-CNN achieved the highest test accuracy among all evaluated models, suggesting that local spectral response patterns between adjacent wavelengths played an important role in distinguishing ZBM and HBBM.

Transformer and CNN-Transformer models were also included as additional deep learning baselines. The Transformer model achieved effective classification performance, but it did not outperform the best convolutional and residual models. CNN-Transformer performed better than the pure Transformer model, indicating that local convolutional feature extraction before global attention-based modeling was beneficial for spectral classification. However, both Transformer-related models showed lower test accuracy than 1D-CNN and 1D-ResNet18. This may indicate that, under the current dataset and binary classification setting, local spectral patterns captured by convolutional and residual architectures were more effective than global self-attention modeling alone. It is also possible that Transformer-based architectures require larger datasets, more extensive hyperparameter optimization, or more task-specific architectural design to fully exploit their long-range dependency modeling ability in spectral classification.

Transforming one-dimensional spectra into two-dimensional GADF images can provide a complementary spectral-structural representation and better express interactions among different wavelengths in an image-like form. The results of the GADF image-based classification models confirmed the feasibility of converting one-dimensional spectra into GADF images for discriminating ZBM and HBBM. However, the GADF transformation did not consistently improve classification performance when used as a standalone input representation. Compared with one-dimensional spectral models, the two-dimensional GADF-based models were generally not clearly superior. In addition, processing GADF images inevitably requires greater computational resources and relatively longer training time. Therefore, for applications where rapid processing and low computational cost are prioritized, direct modeling of one-dimensional spectra may be more efficient. The value of GADF in this study should mainly be understood from the perspective of complementary structural representation. Specifically, GADF images encode inter-band relationships and spectral variation patterns in a two-dimensional form, which can provide structural features for hyperedge construction in SSD-HGNN. Thus, although GADF transformation increases computational complexity, it provides additional structural information that is useful for spectral–structural decoupled hypergraph fusion.

To further mine spectral features from both one-dimensional spectra and GADF images, this study constructed graphs and hypergraphs using different strategies and established GCN and HGNN models for the identification of ZBM and HBBM. The results showed that the HGNN models generally performed better than the corresponding GCN models. This difference may be attributed to the distinct ways in which regular graphs and hypergraphs represent sample relationships. A regular graph describes relationships through pairwise edges, with each edge connecting only two samples. Although multiple pairwise edges can form a local neighborhood, they are treated as separate relationships and cannot explicitly represent the shared association among a group of spectrally similar samples. This limitation is particularly relevant to hyperspectral data, where multiple samples may exhibit similar overall reflectance patterns, local spectral variations, or absorption characteristics and consequently form natural local groups in the spectral feature space. In contrast, a hyperedge can connect multiple related samples simultaneously and represent their shared association as a unified relational group. Hypergraph modeling can therefore capture group-wise spectral consistency and higher-order relationships among samples, rather than describing the data distribution solely through independent pairwise connections. This capability makes hypergraphs more suitable for characterizing the complex local structure of hyperspectral samples, especially in fine-grained classification tasks involving substantial spectral similarity and overlap between categories.

The proposed SSD-HGNN was designed to assign different functional roles to one-dimensional spectral features and two-dimensional GADF structural features within a hypergraph learning framework. Specifically, deep one-dimensional spectral features were used as node attributes to provide discriminative information for propagation, whereas deep GADF image features were used for hyperedge construction to define high-order sample relationships. This design differs from simple feature concatenation, because the two modalities are not merely stacked into a single high-dimensional feature vector. Instead, they are decoupled into node representation and hypergraph topology construction. The experimental results showed that SSD-HGNN achieved competitive overall performance, with stable accuracy, precision, recall, and F1-score on the training, validation, and test sets. The additional precision, recall, and F1-score results were generally consistent with accuracy, indicating that the models did not obtain high accuracy at the expense of clearly imbalanced precision or recall.

It should be noted that the performance advantage of SSD-HGNN over several strong one-dimensional deep learning models was limited. For example, 1D-CNN and 1D-ResNet18 also achieved high test accuracies, indicating that one-dimensional spectral deep learning models were already highly effective for this binary classification task. Although SSD-HGNN did not achieve the highest test accuracy among all evaluated models, it achieved the highest validation accuracy and showed strong overall performance across multiple evaluation metrics. Therefore, the practical value of SSD-HGNN lies not only in its classification accuracy, but also in its ability to integrate one-dimensional spectral features and GADF-derived structural information within a hypergraph learning framework. This provides a competitive multimodal hypergraph strategy for modeling high-order relationships among samples and complementary spectral-structural representations.

The post-hoc interpretability results provide further insight into how SSD-HGNN uses spectral attributes and hypergraph structural information. Spectral-region occlusion showed that the model predictions were particularly sensitive to several localized wavelength intervals rather than being uniformly dependent on the entire spectral range. Among these intervals, 1617.49–1649.78 nm and 1187.00–1219.28 nm produced the largest reductions in the predicted probability of the true class. The identified near-infrared regions are broadly associated with overtone and combination absorptions involving C–H and O–H bonds and may therefore reflect compositional and structural differences between ZBM and HBBM. The sensitivity observed around 1377.13–1409.42 nm may also be associated with O–H-related absorption. These results further support the ability of near-infrared hyperspectral imaging to capture internal compositional differences that may not be readily distinguished from the external appearance of dried slices. Nevertheless, the identified wavelength intervals represent model-based spectral sensitivity and should not be interpreted as direct evidence of specific chemical constituents without targeted chemical measurements.

Hyperedge-removal analysis further confirmed the importance of hypergraph structural information in SSD-HGNN predictions. For 89.25% of the test samples, removing the corresponding inductive hyperedge reduced the predicted probability of the true class, indicating that the structural relationships established between test samples and training reference samples generally provided supportive information for classification. The differences in the magnitude of the probability reductions also indicate that the influence of individual hyperedges varied across test samples. This is reasonable because each inductive hyperedge represents a sample-specific high-order relationship between an unknown sample and its neighboring reference samples in the learned GADF feature space. Consequently, these hyperedges can provide meaningful structural evidence by propagating information among samples with similar inter-band patterns. The results therefore support the role of GADF-derived hypergraph structure as an important complement to one-dimensional spectral representation in SSD-HGNN.

The improvement in representation ability should also be considered together with the increased computational cost introduced by the proposed hypergraph-based framework. Compared with conventional machine learning models and basic deep learning models, HGNN-based models require additional steps, including deep feature extraction, graph or hypergraph construction, incidence matrix calculation, and hypergraph convolution. In particular, SSD-HGNN further combines one-dimensional spectral features with GADF-derived structural features, which increases the complexity of data processing and model computation. Therefore, there is a trade-off between classification performance and processing efficiency. For practical applications, simpler models such as SVC, 1D-CNN, or 1D-ResNet may be more suitable when low computational cost and fast inference are prioritized, whereas SSD-HGNN may be more appropriate when multimodal representation and high-order relationship modeling are considered important.

The graph and hypergraph construction strategies used in this study are suitable for reference-set-based quality-control scenarios. In practical deployment, a new unknown sample can be inserted into an existing reference graph or hypergraph as an unlabeled node and classified according to its feature-similarity relationships with labeled reference samples, rather than constructing an isolated graph for the new sample alone. During validation and test inference in this study, validation and test samples were inserted as unlabeled nodes, and their labels were strictly excluded from graph construction, hyperedge construction, loss calculation, parameter optimization, and model training. Therefore, this strategy did not introduce label leakage. Nevertheless, future studies should further investigate more flexible inductive graph-learning strategies and dynamic graph update mechanisms for practical deployment.

Several limitations should also be acknowledged. First, model performance in this study was evaluated using a fixed stratified training, validation, and test split. Although this setting ensured that all models were compared under identical data conditions, repeated experiments with different random seeds were not conducted. Future work should include repeated random-split experiments, cross-validation, confidence interval estimation, mean ± standard deviation reporting, and appropriate statistical tests, such as McNemar’s test or bootstrap testing, to more comprehensively assess the robustness and statistical significance of performance differences among models. Second, because the commercially sourced dried slices were not traceable to their original bulbs or plants, specimen-level independence across the training, validation, and test sets could not be fully verified. Although each complete slice and its extracted spectrum were assigned to only one subset, different slices originating from the same bulb or plant may potentially have been distributed across different subsets. Future studies should therefore use traceable materials and perform grouped dataset partitioning according to independent plants, bulbs, and acquisition batches to more rigorously avoid potential specimen-level information leakage. Third, although SSD-HGNN demonstrates the feasibility of attribute–topology decoupled spectral–structural fusion, this study did not exhaustively compare it with simpler multimodal fusion strategies, such as direct feature concatenation, score-level fusion, or late decision fusion. Future studies should include these simpler fusion baselines to more comprehensively evaluate the specific contribution of the proposed decoupled hypergraph fusion mechanism. Fourth, all spectral data were obtained from a single experimental dataset using the same hyperspectral imaging device under controlled illumination conditions. Therefore, the robustness and generalizability of SSD-HGNN to spectral noise, variations in illumination intensity or color temperature, and differences among imaging devices have not yet been systematically evaluated. These factors may alter the measured spectral responses and the resulting sample relationships, thereby affecting both spectral feature extraction and hypergraph construction. Future studies will conduct controlled spectral-noise perturbation experiments, collect data under different illumination conditions, and perform external validation using multiple hyperspectral devices and independently acquired datasets. Domain adaptation and spectral calibration transfer strategies will also be investigated to improve the robustness and cross-device generalizability of SSD-HGNN.

Although the post-hoc analyses conducted in this study improved the interpretability of SSD-HGNN, several limitations remain. Spectral-region occlusion and individual hyperedge removal quantify model sensitivity but do not establish causal relationships between specific wavelength intervals, chemical constituents, hypergraph structures, and model predictions. Moreover, individual hyperedge removal does not fully characterize the joint influence of combinations of hyperedges or more complex explanatory sub-hypergraphs. Future studies should therefore further integrate explanatory sub-hypergraph extraction, SHAP or gradient-based feature-attribution methods, and targeted chemical reference measurements to establish a more comprehensive connection among spectral regions, hypergraph structures, and compositional differences between ZBM and HBBM. In addition, future work may include more Fritillaria species, explore hypergraph neural networks based on richer multimodal spectral representations, compare additional lightweight fusion strategies, and further optimize model lightweighting and computational efficiency to better balance classification performance and processing efficiency in practical quality-control applications.

## 5. Conclusions

In this study, hyperspectral imaging combined with machine learning, deep learning, graph neural network, and hypergraph neural network models was used to identify slices of ZBM and HBBM. The results showed that one-dimensional spectral models achieved effective classification performance, indicating that the extracted spectra contained useful discriminative information for distinguishing the two *Fritillaria* species. Among the one-dimensional spectral models, 1D-CNN and 1D-ResNet18 showed strong classification performance, while Transformer and CNN-Transformer also achieved effective results but did not outperform the convolutional and residual models under the current dataset and model settings.

To further explore spectral-structural representation, one-dimensional spectra were converted into two-dimensional GADF images. The GADF-based models achieved effective classification performance, demonstrating the feasibility of transforming spectral sequences into two-dimensional image representations for ZBM and HBBM identification. However, the GADF-based models were not clearly superior to the one-dimensional spectral models when used as standalone input representations. Therefore, GADF images should be regarded as a complementary structural representation rather than a direct replacement for one-dimensional spectra.

The results of GCN and HGNN models showed clear differences. HGNN models generally outperformed GCN models, indicating that learning high-order relationships among spectral samples was more effective than modeling only pairwise sample relationships. In particular, the proposed SSD-HGNN integrated deep one-dimensional spectral features as node attributes and deep GADF image features for hyperedge construction, achieving competitive overall performance among the evaluated models. Although SSD-HGNN did not achieve the highest test accuracy in a single-metric comparison, it achieved the highest validation accuracy among the evaluated models, showed stable performance across accuracy, precision, recall, and F1-score, and demonstrated the feasibility of spectral–structural decoupled hypergraph fusion for Fritillaria species identification.

The computational-cost analysis showed that SSD-HGNN required 16.5999 M parameters, 0.3122 ms per sample, and 414.0625 MB of peak GPU memory, indicating a reasonable balance between computational efficiency and spectral–structural representation capability. These findings demonstrate the potential of hyperspectral imaging combined with hypergraph learning for rapid and nondestructive identification of Fritillaria species and provide a useful reference for related spectral analysis tasks.

In future work, more *Fritillaria* species will be included, and more suitable hypergraph construction strategies for spectra and GADF images will be further investigated. In addition, spectral feature transformation methods and multimodal spectral representations should be further explored. Simpler multimodal fusion strategies, such as feature-level concatenation and decision-level fusion, should also be compared to more comprehensively evaluate the contribution of the proposed decoupled hypergraph fusion mechanism. Model interpretability should also be further strengthened through wavelength-contribution analysis and other post-hoc interpretation methods. Meanwhile, future work will focus on lightweight backbone design, feature dimensionality reduction, and more efficient hypergraph construction to further reduce computational costs and improve the applicability of SSD-HGNN in resource-constrained and real-time quality-control scenarios.

## Figures and Tables

**Table 1:** Sample distribution of ZBM and HBBM.

	Total	Training	Validation	Test
ZBM	5162	3440	860	862
HBBM	5890	3926	983	981
Total	11,052	7366	1843	1843

**Table 2:** Classification performance of different models based on one-dimensional spectral data for discriminating ZBM and HBBM.

Methods	Train				Val				Test			
	Accuracy	Precision	Recall	F1-Score	Accuracy	Precision	Recall	F1-Score	Accuracy	Precision	Recall	F1-Score
LR	0.9187	0.9196	0.9171	0.9181	0.9251	0.9260	0.9237	0.9246	0.9093	0.9098	0.9081	0.9088
SVC	0.9705	0.9710	0.9699	0.9704	0.9457	0.9465	0.9446	0.9454	0.9408	0.9417	0.9396	0.9404
XGBoost	0.9438	0.9449	0.9424	0.9434	0.8887	0.8892	0.8872	0.8880	0.8740	0.8746	0.8723	0.8732
1D-CNN	0.9580	0.9586	0.9572	0.9578	0.9435	0.9444	0.9424	0.9432	0.9479	0.9483	0.9470	0.9476
Transformer	0.9274	0.9279	0.9262	0.9269	0.9180	0.9182	0.9170	0.9176	0.9159	0.9177	0.9138	0.9152
CNN-Transformer	0.9511	0.9510	0.9507	0.9509	0.9414	0.9412	0.9410	0.9411	0.9349	0.9350	0.9341	0.9345
1D-ResNet18	0.9810	0.9700	0.9678	0.9687	0.9463	0.9522	0.9499	0.9508	0.9452	0.9450	0.9420	0.9431
1D-ResNet34	0.9722	0.9680	0.9664	0.9671	0.9452	0.9518	0.9501	0.9508	0.9441	0.9427	0.9399	0.9410
1D-GCN	0.8938	0.8939	0.8938	0.8937	0.8931	0.8931	0.8931	0.8931	0.8789	0.8790	0.8789	0.8788
RawSpec-RawSpec-HGNN	0.9499	0.9501	0.9492	0.9496	0.9311	0.9304	0.9317	0.9309	0.9186	0.9180	0.9189	0.9183
ResSpec-RawSpec-HGNN	0.9893	0.9894	0.9890	0.9892	0.9533	0.9535	0.9527	0.9531	0.9414	0.9424	0.9401	0.9410
RawSpec-ResSpec-HGNN	0.9431	0.9433	0.9424	0.9428	0.9083	0.9172	0.9037	0.9067	0.8947	0.9049	0.8897	0.8927
ResSpec-ResSpec-HGNN	0.9890	0.9892	0.9887	0.9889	0.9533	0.9534	0.9528	0.9531	0.9425	0.9433	0.9413	0.9421

Note: RawSpec-RawSpec-HGNN denotes a hypergraph neural network in which both node attributes and hyperedges are constructed based on raw one-dimensional spectral data. ResSpec-RawSpec-HGNN denotes a model in which node attributes are derived from deep features extracted by 1D-ResNet, while hyperedges are constructed based on raw spectral similarity. RawSpec-ResSpec-HGNN denotes a model using raw spectral node attributes and hyperedges induced by deep 1D-ResNet features. ResSpec-ResSpec-HGNN denotes a model in which deep 1D-ResNet features are used for both node attributes and hyperedge construction.

**Table 3:** Classification performance of different models based on GADF images for discriminating ZBM and HBBM.

Methods	Train				Val				Test			
	Accuracy	Precision	Recall	F1-Score	Accuracy	Precision	Recall	F1-Score	Accuracy	Precision	Recall	F1-Score
2D-CNN	0.9606	0.9723	0.9708	0.9715	0.9381	0.9334	0.9324	0.9329	0.9229	0.9346	0.9334	0.9339
2D-ResNet18	0.9868	0.9876	0.9861	0.9868	0.9332	0.9352	0.9313	0.9327	0.9321	0.9345	0.9300	0.9315
2D-ResNet34	0.9947	0.9947	0.9947	0.9947	0.9273	0.9270	0.9269	0.9269	0.9316	0.9318	0.9307	0.9312
2D-GCN	0.9362	0.9360	0.9358	0.9359	0.8979	0.8976	0.8973	0.8974	0.8893	0.8892	0.8882	0.8886
RawGADF-RawGADF-HGNN	0.9856	0.9862	0.9850	0.9855	0.9327	0.9322	0.9326	0.9324	0.9224	0.9219	0.9222	0.9221
ResGADF-RawGADF-HGNN	0.9940	0.9943	0.9937	0.9940	0.9349	0.9351	0.9340	0.9345	0.9273	0.9278	0.9261	0.9268
RawGADF-ResGADF-HGNN	1.0000	1.0000	1.0000	1.0000	0.9349	0.9344	0.9349	0.9346	0.9245	0.9243	0.9241	0.9242
ResGADF-ResGADF-HGNN	0.9950	0.9951	0.9948	0.9950	0.9349	0.9348	0.9343	0.9345	0.9251	0.9255	0.9241	0.9246
**SSD-HGNN**	**0.9848**	**0.9847**	**0.9847**	**0.9847**	**0.9533**	**0.9528**	**0.9535**	**0.9531**	**0.9441**	**0.9438**	**0.9439**	**0.9438**

Note: RawGADF-RawGADF-HGNN denotes a hypergraph neural network in which both node attributes and hyperedges are constructed based on raw GADF features. ResGADF-RawGADF-HGNN denotes a model in which node attributes are derived from deep GADF features extracted by 2D-ResNet, while hyperedges are constructed based on raw GADF feature similarity. RawGADF-ResGADF-HGNN denotes a model using raw GADF node attributes and hyperedges induced by deep GADF features. ResGADF-ResGADF-HGNN denotes a model in which deep GADF features are used for both node attributes and hyperedge construction. SSD-HGNN denotes the proposed spectral–structural decoupled hypergraph neural network, in which deep 1D spectral features are used as node attributes and deep GADF image features are used for hyperedge construction. The best-performing results for each task are highlighted in bold.

**Table 4:** Computational costs of the comparison models and the proposed SSD-HGNN.

Model	Params (M)	Inference Time/Sample (ms)	Peak GPU Memory (MB)
1D-CNN	2.3081	0.0112	19.6162
Transformer	0.5626	0.0530	125.0449
CNN-Transformer	1.2449	0.0657	141.3687
1D-ResNet18	3.8449	0.0246	35.2065
1D-ResNet34	7.2193	0.0432	48.1167
1D-GCN	0.0520	0.0524	52.5659
RawSpec-RawSpec-HGNN	0.3674	0.2545	74.6455
ResSpec-RawSpec-HGNN	4.3720	0.2973	100.8140
RawSpec-ResSpec-HGNN	4.2123	0.3679	88.6924
ResSpec-ResSpec-HGNN	4.3720	0.3677	101.0977
2D-CNN	11.1805	0.1752	237.0396
2D-ResNet18	11.1775	0.4258	394.3159
2D-ResNet34	21.2857	0.5258	433.6636
2D-GCN	5.1204	1.3719	1452.2979
RawGADF-RawGADF-HGNN	20.7450	1.2968	5712.8862
ResGADF-RawGADF-HGNN	11.7036	1.2995	395.9746
RawGADF-ResGADF-HGNN	31.9215	0.7083	5753.8350
ResGADF-ResGADF-HGNN	11.7036	0.3005	395.4570
**SSD-HGNN**	16.5999	0.3122	414.0625

## Data Availability

The original contributions presented in this study are included in the article. Further inquiries can be directed to the corresponding author.
